# N-deglycosylation targeting chimera (DGlyTAC): a strategy for immune checkpoint proteins inactivation by specifically removing N-glycan

**DOI:** 10.1038/s41392-025-02219-6

**Published:** 2025-04-28

**Authors:** Li Li, Jiajia Wu, Weiqian Cao, Wei Zhang, Qi Wu, Yaxu Li, Yanrong Yang, Zezhi Shan, Zening Zheng, Xin Ge, Liang Lin, Ping Wang

**Affiliations:** 1 Tongji University Cancer Center, Shanghai Tenth People’s Hospital, School of Medicine, Tongji University, Shanghai, China; 2 State Key Laboratory of Chemical Biology, Shanghai Institute of Organic Chemistry, Chinese Academy of Sciences, Shanghai, China; 3 Institutes of Biomedical Sciences, Fudan University, Shanghai, China; 4 Shanghai Frontiers Science Center of Nanocatalytic Medicine, School of Medicine, Tongji University, Shanghai, China; 5 Department of Colorectal Surgery, Fudan University Shanghai Cancer Center, Shanghai, China; 6 Department of Oncology, Shanghai Medical College, Fudan University, Shanghai, China

**Keywords:** Drug development, Immunological techniques

## Abstract

Among the leading methods for triggering therapeutic anti-cancer immunity is the inhibition of immune checkpoint pathways. N-glycosylation is found to be essential for the function of various immune checkpoint proteins, playing a critical role in their stability and interaction with immune cells. Removing the N-glycans of these proteins seems to be an alternative therapy, but there is a lack of a de-N-glycosylation technique for target protein specificity, which limits its clinical application. Here, we developed a novel technique for specifically removing N-glycans from a target protein on the cell surface, named deglycosylation targeting chimera (DGlyTAC), which employs a fusing protein consisting of Peptide-N-glycosidase F (PNGF) and target-specific nanobody/affibody (Nb/Af). The DGlyTAC technique was developed to target a range of glycosylated surface proteins, especially these immune checkpoints—CD24, CD47, and PD-L1, which minimally affected the overall N-glycosylation landscape and the N-glycosylation of other representative membrane proteins, ensuring high specificity and minimal off-target effects. Importantly, DGlyTAC technique was successfully applied to lead inactivation of these immune checkpoints, especially PD-L1, and showed more potential in cancer immunotherapy than inhibitors. Finally, PD-L1 targeted DGlyTAC showed therapeutic effects on several tumors in vivo, even better than PD-L1 antibody. Overall, we created a novel target-specific N-glysocylation erasing technique that establishes a modular strategy for directing membrane proteins inactivation, with broad implications on tumor immune therapeutics.

## Introduction

Within the tumor microenvironment, cancer cells express inhibitory ligands, through which cancer cells function to promote tumor immune tolerance and evade immune system eradication.^1^ Recently, the pharmacological modulation of these pathways, termed immune checkpoint (IC) therapy, has been extensively investigated and developed as novel immunotherapeutic agents for cancer treatment.^2^ In the last decade, the use of immune checkpoint inhibitors (ICIs) in immunotherapy has dramatically improved the treatment outcomes for various cancer types.^3^ The clinical use of ICIs that target CTLA4, PD-1, and PD-L1 have revolutionized cancer treatment, a milestone recognized by the awarding of the 2018 Nobel Prize in Medicine and Physiology.^4^

ICIs, as a class of monoclonal antibodies, possess the capability to bind with high specificity to ICs on the surface of cells. Each monoclonal antibody molecule has the potential to bind to a single epitope on the target protein. Given this, maintaining a sufficient inhibitor concentration at the desired site of action for the required duration is crucial to achieving their immunomodulatory functions against cancer.^5^ The above issues are felt most keenly when designing inhibitors, so other modalities are being used to circumvent such limitations. The most representative one is Proteolysis Targeting Chimeras (PROTACs), which as catalytic agents facilitate the recruitment of target proteins to E3 ubiquitin ligases, thereby tagging the target proteins for ubiquitination and subsequent proteasomal degradation. Unlike conventional inhibitors that suppress protein activity through sustained binding, PROTACs induce targeted protein degradation, allowing them to achieve therapeutic effects at lower concentrations with degrader recycling.^6^ Furthermore, similar techniques, such as Lysosome-targeting chimeras (LYTACs) and antibody-based PROTACs (AbTACs),^7^ springs up recently for targeting out membrane proteins. These technologies depend on specific natural degradation processes, making them difficult to develop a straightforward and widely used approach applied for all cell types. ICs normally require post-translational modifications (PTMs), such as phosphorylation and glycosylation,^8^ to enable a broad range of functions. N-glycosylation is one of the most extensive PTMs on the extracellular surface of ICs, which adds glycan moieties to asparagine side-chains of proteins. N-glycosylation plays a prominent role in common biological process, such as cellular differentiation, cell–cell communication, protein stability, tumorigenesis and immune response.^9–13^ Approximately 80% of cell-surface proteins are N-glycosylated, and abnormal glycosylation of tumor cells can interfere with immune cell recognition, weakening their anti-tumor effects. Therefore, blocking N-glycosylation on the surface of tumor cells can enhance the cytotoxicity of CAR-T cells against malignant tumors.^14^

N-glycosylation plays a prominent and multifaceted role in the immune response, particularly in the regulation of a series of immune checkpoint (IC) pairs, such as PD-L1 and PD-1, PVR and TIGIT, CTLA4 and B7-1, and CD70 and CD27.^15^ While, the functions of N-glycosylation on a bunch of ICs remain unstudied. We propose that by fusing a target recognition element to Peptide-N-glycosidase F (PNGF), a general N-glycan eraser which is widely used in removing N-glycans on the cell surfaces and intact proteins,^16^ to specifically erase the N-glycans of target proteins. This strategy not only expands the toolkit for membrane protein manipulation but also provides a novel method for studying and therapeutically targeting glycosylation-dependent processes. Moreover, similar to other advanced protein-targeting technologies like PROTACs (Proteolysis Targeting Chimeras), LYTACs (Lysosome-Targeting Chimeras), and AbTACs (Antibody-Based PROTACs), which have emerged recently as powerful tools for targeting membrane proteins, these approaches leverage the catalytic and recyclable nature of the fusion complex to achieve precise and efficient modulation of protein function. These technologies share a common principle of using bifunctional molecules to bridge target proteins with effector mechanisms, such as degradation or modification, offering a high degree of specificity and potency.

Here, we developed a technique that can specifically erase the glycans from a target protein on live cells through combination of PNGF with the specific nanobody/affibody (Nb/Af) of the target protein, in which the complex was termed Deglycosylation Targeting Chimera (DGlyTAC). We engineered specific DGlyTACs for various proteins, achieving targeted removal of N-glycans from the desired proteins, including growth factor receptors (EGFR, HER2, IGF1R) and ICs (CD24, CD47, PD-L1), while exerting minimal influence on the overall N-glycosylation landscape. DGlyTAC technique is a general strategy for in situ elimination N-glycans to disrupt the binding of CD24/Siglec-10, as well as the affinity of PD-L1/PD-1. This property not only enhances their therapeutic potential but also minimizes off-target effects and reduces the risk of toxicity, making them highly suitable for clinical applications. By leveraging the specificity of target recognition elements and the enzymatic activity of PNGF, this approach could revolutionize the study and therapeutic targeting of glycosylated immune checkpoint proteins. It opens new avenues for understanding the role of N-glycosylation in immune regulation and provides a versatile platform for developing next-generation immunotherapies, particularly in cancer treatment, where immune checkpoint modulation is a key strategy. This innovative technique could also be extended to other diseases where glycosylation plays a critical role, further broadening its impact on biomedical research and therapeutic development.

## Results

### Specific removal of N-glycans from overexpressed eGFP-CD24 using DGlyTAC

**Fig. 1:**
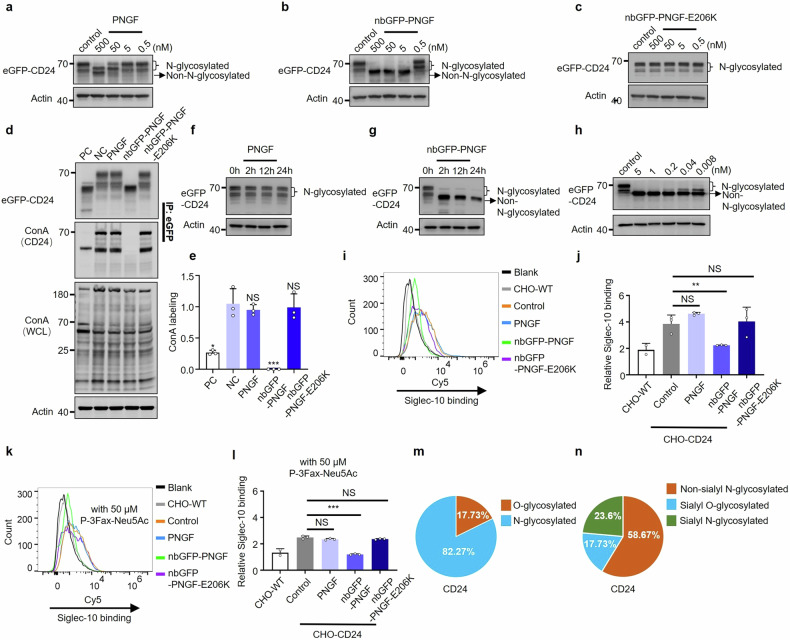
Design of DGlyTAC nbGFP-PNGF for protein-specific de-N-glycosylation on eGFP-CD24. Western blot analyzed the effect on the expression and glycosylation of eGFP-CD24 which overexpression on CHO cell lines after the treatment of different concentrations of PNGF (**a**), nbGFP-PNGF (**b**), and nbGFP-PNGF-E206K (**c**) for 2 h. (**d**) eGFP-CD24 was enriched by anti-eGFP beads and analyzed by immunoblotting to reveal protein levels (anti-GFP) and N-glycosylation level (ConA). CHO-CD24 whole cell lysate (WCL) was treated with 1 μM PNGF (as positive control, PC), PBS (as nagative control, NC), 0.05 nM PNGF, nbGFP-PNGF, and nbGFP-PNGF-E206K for 24 h, we proceeded with the labeling of N-glycans using ConA-Biotin. (**e**) Quantitation of N-glycosylation level on eGFP-CD24 calculated from (**d**). (The box and error bars represent the mean ± SEM. Independent experiment = 3, and a representative data was shown; ‘NS’ means not significant; ‘*’ means *p* < 0.05; ‘**’ means *p* < 0.01; ‘***’ means *p* < 0.001) (**f**, **g**) Change of eGFP-CD24 protein level via treatment from 5 nM PNGF and nbGFP-PNGF at different time points. **h** Immunoblotting of eGFP-CD24 resulted from cells exposed to low concentrations of nbGFP-PNGF for 24 h. **i**, **k** Treated CHO-CD24 with or without 50 µM sialyltransferase inhibitor P-3Fax-Neu5Ac (deleting sialic acids on CHO-CD24), then incubated with 5 nM PNGF and nbGFP-PNGF for 2 hours, and flow cytometry measuring Siglec-10 binding on the membrane of eGFP-CD24. **j**, **l** Quantitation of the CD24/Siglec-10 binding from i and k, respectively. The relative values are results divided by blank. (The box and error bars represent the mean ± SEM. Independent experiment = 3, and a representative data was shown; ‘NS’ means not significant; ‘*’ means *p* < 0.05; ‘**’ means *p* < 0.01; ‘***’ means *p* < 0.001) **m**, **n** Assessment about the contributions of sialyl modification and N/O-glycosylation on CD24/Siglec-10 interaction in the CHO-CD24 cells analyzed from (**j**, **l**)

To prove the concept, we first applied our strategy to a tag-fused protein with fewer N-glycosylation sites and a smaller molecular weight. Cluster of differentiation 24 (CD24), a glycoprotein contain two N-glycosylation sites and anchors to glycosyl-phosphatidyl-inositol (GPI),^17^ we first fused CD24 with enhanced green fluorescent protein (eGFP) at the N-terminus as a tag for targeting. We transfected pcDNA3.1-eGFP-CD24 plasmid into CHO cells (termed CHO-CD24) which lack of CD24 expression. We then confirmed that treatment with 500 nM PNGF for 2 h in serum-free RPMI medium removed nearly all N-linked glycans from eGFP-CD24 and resulted in the appearance of protein bands with lower molecular weights (Fig. 1a). To improve specificity, we engineered PNGF which was fused with an N-terminal nanobody against eGFP (nbGFP) to generate DGlyTAC nbGFP-PNGF. De-N-glycosylation of eGFP-CD24 was completely achieved with 5 nM nbGFP-PNGF (PNGF fused to nanobody with targeting capability), whereas 500 nM PNGF (lack of targeting capability) could not (Fig. 1a, b)—in other words, nbGFP-PNGF required only one-hundredth of the concentration of PNGF to obtain optimal results. In parallel, mutation of PNGF at catalytic residue 206 from glutamic acid to lysine rendered an inactive enzyme (nbGFP-PNGF-E206K), which was served as a negative control (Fig. 1c). We also proved the same complete de-N-glycosylation of nbGFP-PNGF on eGFP-CD24 in both cell lysis and live cells (Supplementary Fig. 1a). The remarkable difference between PNGF and nbGFP-PNGF was because of a high binding affinity of nbGFP to eGFP (Kd = 5.58 ± 2.73 nM, Supplementary Fig. 1b).

To further prove the specificity of nbGFP-PNGF, we used mannose specific lectin Concanavalin A (ConA)-Biotin to detect mannose residues in N-glycans and represent the level of N-glycosylation.^18^ Treatment with nbGFP-PNGF exhibited completed removal of N-glycans on eGFP-CD24, while its effect on the global N-glycosylation levels was negligible (Fig. 1d, e). Moreover, nbGFP-PNGF did not significantly reduce the N-glycosylation level of H2-K1 (MHC class I heavy chain precursor) on CHO cells, which could be de-N-glycosylated with high concentration of PNGF (Supplementary Fig. 1h-i). Furthermore, we also explored the effects of the enzyme on eGFP-CD24 protein level at different time points. Treatment with 5 nM nbGFP-PNGF, but not PNGF, for more than 2 h could compromise the CD24 quantity in serum-free RPMI medium because of the removal of N-glycans (Fig. 1f, g). It was noteworthy that low concentrations of nbGFP-PNGF also worked well with extended processing time (like 24 h) in medium containing fetal bovine serum (FBS) (Fig. 1h), indicating that serum does not significantly affect the efficiency of enzymatic de-N-glycosylation. Due to the high efficiency of nbGFP-PNGF, 0.008 nM enzyme de-N-glycosylated the majority of eGFP-CD24. Therefore, nbGFP-PNGF is employed to selectively remove N-glycans of eGFP-CD24 on the cell surface while minimally affecting the global N-glycosylation landscape.

### Harnessing macrophages through the de-N-glycosylation of CD24 and CD47

Siglec-10, an IC, is capable of recognizing both surface proteins (e.g., CD24) and surface sialic acids.^19^ Upon binding of CD24 to Siglec-10, an inhibitory signaling cascade occurs to prevent macrophage phagocytosis and promote tumor growth.^19^ We next tested whether de-N-glycosylated CD24 could also bind to Siglec-10. Our results showed that de-N-glycosylated CD24 lost most of the interaction with Siglec-10 (Fig. 1i, j, m, Supplementary Fig. 1c), suggesting that N-glycans on CD24 are essential for binding to Siglec-10. It has been reported that Siglec-10 interacts with highly sialylated form of CD24.^20^ Meanwhile, deletion of CD24 nearly abolished Siglec-10 binding in absence of surface sialylation, indicating that CD24 is the major protein binder of Siglec-10 ^20^. Moreover, Siglecs also specifically interact with sugar chains via the sialic acid linkage, and consequently N-glycans of complex structure can be recognized and linked to Siglec-10 ^21^. We also evaluated the contribution of sialic acids to CD24 with Siglec-10 binding through the use of P-3Fax-Neu5Ac (a global sialyltransferase inhibitor^22^) on CHO-WT and CHO-CD24 cells, and a significant decrease in Siglec-10 interaction of all groups was observed (Fig. 1k, l). As expected, no Siglec-10 binding was observed in CHO-WT with P-3Fax-Neu5Ac treatment. Unexpectedly, a significant decrease of Siglec-10 interaction was also observed via the treatment of nbGFP-PNGF after P-3Fax-Neu5Ac treatment (Fig. 1k, l, Supplementary Fig. 1d–f), indicating that even without sialic acid modification, the N-glycosylation on CD24 still participates in the binding of CD24/Siglec-10. Overall, in CHO-CD24 cells, N-glycans accounted for more than 82% of the CD24/Siglec-10 interaction, and more than a half of the CD24/Siglec-10 interaction was from non-sialyl N-glycosylated CD24 (Fig. 1m, n, Supplementary Fig. 1g), indicating the significance of N-glycosylation on CD24 in CD24/Siglec-10 interaction.

**Fig. 2:**
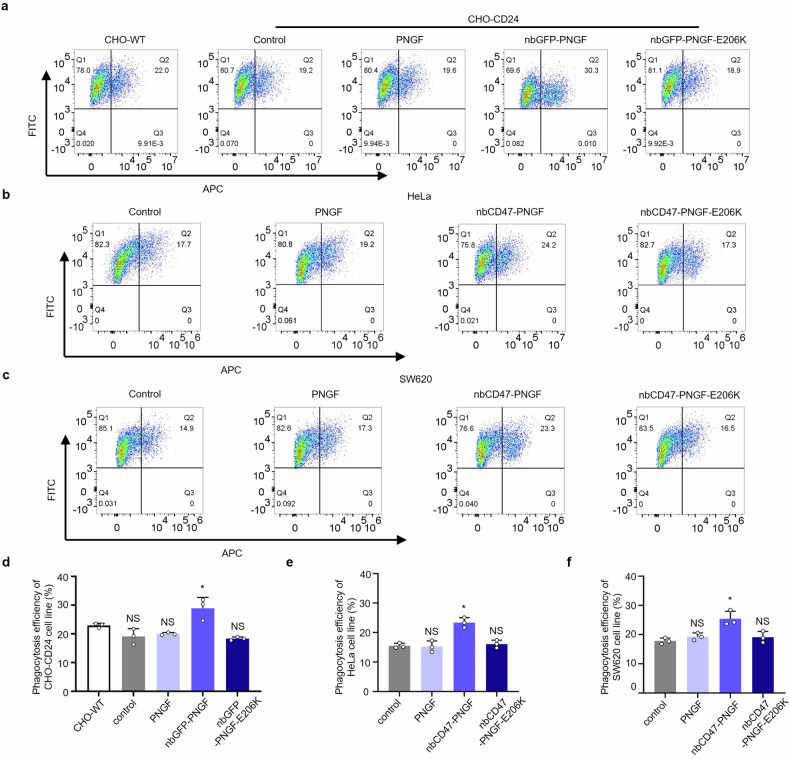
In vitro assessment of the effects of DGlyTAC on phagocytosis. (**a**–**c**) In vitro phagocytosis assays by incubating bone marrow-derived macrophages (BMDMs) with different types of cells. The macrophages were stained with allophycocyanin (APC), CHO-CD24, HeLa, and SW620 cells were labeled with green (FITC). Representative flow-cytometry plots portraying the phagocytosis of cells treated with PNGF, Nb/Af-PNGF, and Nb/Af-PNGF-E206K.**d–f** Quantification of phagocytosis efficiency calculated from (**a**–**c**). (The box and error bars represent the mean ± SEM. Independent experiment = 3, and a representative data was shown; ‘NS’ means not significant; ‘*’ means *p* < 0.05)

Next, we performed in vitro phagocytosis assay by incubating bone marrow-derived macrophages (BMDMs) with either CHO-CD24 cells or CHO-WT cells as control. As expected, we found that nbGFP-PNGF increased phagocytosis by approximately 0.5-fold compared with that of control (Fig. 2a, d), indicating that de-N-glycosylation of CD24 significantly increased the phagocytosis by macrophages. We also used cytochalasin D (decrease the polymerization and depolymerization of actin, an inhibitor of phagocytosis),^23^ to present that the phagocytosis is not fake rather than two cells stuck together (Supplementary Fig. 2d–g).

Another well-known IC pair is CD47-SIRPα. The highly glycosylated portion of CD47 is also essential for its interaction with ligands like integrins.^24^ N-glycosylation sites on CD47 are responsible for keeping the interaction interface reachable.^25^ The high-affinity binding domain from SIRPα was made as a nanobody for CD47 (nbCD47).^26^ Thus, we fused PNGF to nbCD47 to generate DGlyTAC nbCD47-PNGF. Different kinds of cancer cells (MDA-MB231, HeLa, and SW620) were partially deglycosylated by PNGF or nbCD47-PNGF for 24 h in serum-free RPMI medium (Supplementary Fig. 2a–c and 3m). Using nbCD47-PNGF, bands shifted to smaller sizes with partial N-glycosylation erased. Next, we performed in vitro phagocytosis assay, in which cells were incubated with PNGF, nbCD47-PNGF or nbCD47-PNGF-E206K. There existed a significant increase in phagocytosis upon treatment of DGlyTAC nbCD47-PNGF rather than PNGF or the inhibitor competitor nbCD47-PNGF-E206K in HeLa and SW620 cells (Fig. 2b, c, e, f).

Our results showed that de-N-glycosylation of both CD24 and CD47 significantly increased the phagocytosis by macrophages, indicating that N-glycosylation accounts for the interactions between CD24 and Siglec-10, as well as CD47 and SIRPα.

### Expanding the target protein scopes of DGlyTACs

Inspired by these results, we were encouraged to expand the target proteins of DGlyTACs, to a more abundant cell surface protein type—growth factor receptors, which are well-studied with a series of affibodies and nanobodies. Furthermore, N-glycosylation plays a critical role in the extensive expression of growth factor receptors on the cell surface, facilitating their accurate localization on the cell membrane.^27^

We chose IGF1R as our initial investigated growth factor receptor. IGF-1 is regulated by the tyrosine kinase receptor IGF-1R which is synthesized as a single polypeptide precursor.^28^ Activated IGF-1R is proteolytically cleaved into several chains with different weights (α subunit 130 KDa and β subunit 97 KDa) that form a tetramer (α-β-β-α).^28^ Each α and β subunit contains 11 and 5 N-glycosylation sites, respectively.^28^ A number of studies have shown that IGF-1R is accurately matured and transported to the cell surface when its N-glycosylation is appropriately maintained.^29,30^ The affibody molecule afIGF1R (Kd = 2.3 nM), which binds to the hormone interaction site in IGF-1R, efficiently recognizes IGF1R-β in various contexts.^31^ After partially de-N-glycosylation by PNGF or afIGF1R-PNGF in serum-free RPMI medium for 2 h in different kinds of cancer cells (MCF7, HepG2 and SK-BR-3), the bands shifted to the smaller size (~70 kDa) compared to that of control group. The afIGF1R-PNGF showed higher ability than PNGF in removal of N-glycans from IGF1R-β (Supplementary Fig. 3a–c, l).

Similar strategy was applied to epidermal growth factor receptor (EGFR) and one of its homologous receptors HER2. The EGFR is one of the most studied growth factor receptors, which can form one type of homodimer and three types of heterodimers constituted by EGFR and HER2, thus regulating critical cellular processes and serving as an important target for cancer therapy. In order to understand the complex functions of EGFR/HER2 on the membrane, structural analysis is deeply required. Due to heterodimerization, these receptors can be activated by a greater number of ligands and also capable of initiating various signaling pathways.^32^ N-glycosylation plays an important role in membrane interaction, structure establishment, and dimer formation.^27^ We fused affibody molecule of afEGFR (Kd = 2.8 nM) and afHER2 (Kd = 3 nM) to PNGF, respectively.^33,34^ The protein bands of EGFR and HER2 were shifted to a smaller size in the afEGFR-PNGF and afHER2-PNGF groups compared to that in control group (Supplementary Fig. 3d–i), but not as small as the non-N-glycosylated forms (Supplementary Fig. 3j, k). EGFR and HER2 are large proteins with substantial molecular weights, which complicates the ability of DGlyTACs to access all glycosylation sites on these proteins. Additionally, PNGF preferentially cleaves N-glycosylation sites found in loop regions of the proteins. This may because the N-glycosylation sites in EGFR and HER2 always locate on the α-helix/β-helix foldings, which may lead to the suboptimal use of afEGFR-PNGF and afHER2-PNGF for editing the N-glycans. Additionally, EGFR and HER2 proteins have a large molecular weight and many N-glycosylation sites. Even using a high concentration (1 μM) of PNGF, only one or two N-glycan sites can be removed, indicating that other glycosylation sites are not accessible to PNGF. Glycosylation of cell surface growth factors is a sophisticated and crucial biological process that markedly influences cell function and behavior. Researches into glycosylation of these receptors not only contribute to better understanding of relevant disease mechanisms but also break a new avenue for future therapeutics.

DGlyTACs can specifically remove N-glycans from various target proteins on different cells. This strategy is not limited by proteins or cell types, specifically for exploring the importance of the N-glycans on glycoproteins, aiding in the development of more practical antibody-enzyme conjugates for targeted therapies.

### DGlyTAC specifically erasing the N-glycans of PD-L1 and blocking its binding to PD-1

In tumor immunity, T cells and macrophages both play pivotal roles, but their functions and effects are distinct. T cells are central to tumor immunotherapy, being directly involved in the recognition and elimination of tumor cells. The function of T cells is particularly critical in ICI therapy, making them a focal point in tumor immunity.^35^ Consequently, we have been investigating the impact of N-glycosylation modifications on ICs and their influence on T cell function.

**Fig. 3:**
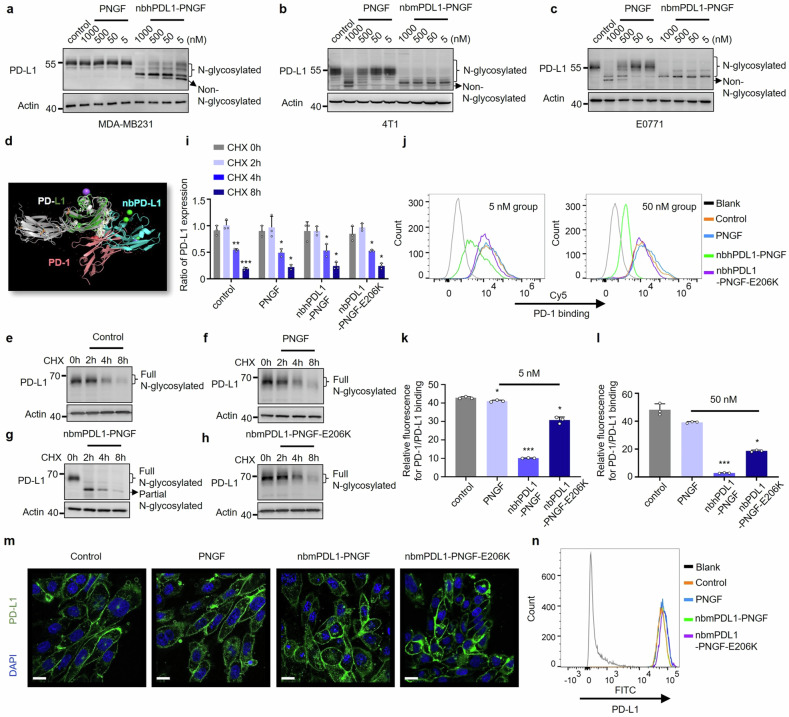
N-glycosylation showing no impact on PD-L1 stability but vital for its interaction to PD-1. **a**–**c** Immunoblotting of PD-L1 after 2-hour incubation with concentration gradient of PNGF and nbhPDL1-PNGF/nbmPDL1-PNGF for MDA-MB231, 4T1, and E0771 cell lines. **d** Structure alignment of PD-1 and NbPD-L1 with the same binding site on PD-L1 (PDB 4zqk and 5jds). PD-L1 is depicted in white and green, PD-1 in pink, and nbhPD-L1 in blue. **e**, **h** Immunoblots showing protein levels of PD-L1 for E0771 cells treated with PNGF, nbmPDL1-PNGF or nbmPDL1-PNGF-E206K as well as CHX during indicated time points. **i** Quantitation of the PD-L1 level calculated from (**e**–**h**). (The box and error bars represent the mean ± SEM. Independent experiment = 3, and a representative data was shown; ‘NS’ means not significant; ‘*’ means *p* < 0.05; ‘**’ means *p* < 0.01; ‘***’ means *p* < 0.001) (**j**) After MD-MB231 cells were treated with 5 nM or 50 nM PNGF, nbhPDL1-PNGF, and nbhPDL1-PNGF-E206K for 24 h, PD-1 binding was measured using flow cytometry. **k**, **l** Quantitation of the PD-1/PD-L1 binding calculated from (**j**). (The box and error bars represent the mean ± SEM. Independent experiment = 3, and a representative data was shown; ‘NS’ means not significant; ‘*’ means *p* < 0.05; ‘**’ means *p* < 0.01; ‘***’ means *p* < 0.001). **m** Immunofluorescence staining of PD-L1 (green) after E0771 cells were treated with PNGF, nbhPDL1-PNGF, and nbhPDL1-PNGF-E206K. DAPI (blue) was used to stain the nucleus. The scale bars in the lower left corner of the image represent 50 μm. **n** The representative flow cytometry presents the PD-L1 level after the same treatment as m

It has been reported that tumor cells evade immune surveillance by altering glycosylation patterns of their surface proteins.^36^ Glycosylation is crucial for the associations between immune ligands and their corresponding receptors.^36^ To enhance immune efficiency toward tumors, related researches were conducted through compromising the recognition of surface structures dominated by their glycosylation on tumor cells, which were already evidenced by a reduction in tumor escape via application of counteracting antibodies specifically directed to glycosylation complex.^36^ It is well-established that N-glycosylation is required for the interaction between PD-L1 and PD-1.^15^ The PD-L1, a famous immune inhibitory ligand in cancer cell, exhibited a most significant loss in receptor binding after its de-N-glycosylation.^15^ Thus, we focused on protein-specific de-N-glycosylation for PD-L1, in which human PD-L1 nanobody (nbhPDL1) and mouse PD-L1 nanobody (nbmPDL1) were utilized to generate DGlyTACs nbhPDL1-PNGF and nbmPDL1-PNGF.^37^ A significant reduction in the molecular weight of PD-L1 were observed in the presence of nbhPDL1-PNGF or nbmPDL1-PNGF (Fig. 3a–c, Supplementary Fig. 4a–c, g). Complete de-N-glycosylation or nearly complete de-N-glycosylation was observed (Fig. 3a–c, Supplementary Fig. 3n and 5a–c). Nevertheless, PNGF seemed ineffective for human PD-L1 de-N-glycosylation (Fig. 3a). Probing for N-glycosylation on PD-L1 following immunoprecipitation, we revealed that nbhPDL1-PNGF removed nearly all N-glycans of PD-L1 while PNGF or nbhPDL1-PNGF-E206K was not (Supplementary Fig. 4d). Treatment of nbhPDL1-PNGF had a negligible effect on global cellular N-glycosylation level (Supplementary Fig. 4d–e).

NbPDL1-PNGF share the same binding sites as PD-1 and thus blocking the PD-1 and PD-L1 interaction (Fig. 3d). We then assessed the stability of PD-L1 by cycloheximide (CHX, MedChemExpress, Cat# HY-12320) treatment, which can distort measurements of protein translation efficiency,^38^ combined with de-N-glycosylation of PD-L1 by nbhPDL1-PNGF. Our data showed that de-N-glycosylation had no effect on the stability of PD-L1 (Fig. 3e–i). To further confirm whether the cell membrane stability (that present the half-life period of membrane proteins to be degraded in the lysosomal pathway) of PD-L1 depends on N-glycosylation, confocal imaging and flow cytometry were conducted and showed little change in PD-L1 protein level on the membrane of E0771 cells with or without nbmPDL1-PNGF (Fig. 3m, n). Next, we tested whether de-N-glycosylated PD-L1 could also bind to PD-1. In comparison with the PBS as control, PNGF did not prevent PD-L1 from its association with PD-1 (Fig. 3j–l). In contrast, DGlyTAC nbhPDL1-PNGF exhibited a significant disturbance on PD-L1/PD-1 binding in a dose-dependent manner, which was much better to that of the inhibitor competitor nbhPDL1-PNGF-E206K (Fig. 3j–l). As data shown in Fig. 3k, l, nbhPDL1-PNGF markedly decrease the binding between PD-1 and PD-L1 ( ~ 16-fold) at 50 nM, but it was only about 2.5-fold when treated with nbhPDL1-PNGF-E206K at the same concentration.

In conclusion, DGlyTAC nbhPDL1-PNGF can act as a PD-L1 specific glycosidase, which erase the N-glycans on PD-L1 as well as its binding to PD-1. Our results also showed that DGlyTAC does not influence the cell membrane stability of PD-L1, as well the expression rate of PD-L1. Such dual actions make DGlyTAC nbhPDL1-PNGF not only effectively eliminate the interaction between PD-L1 and PD-1, but also present a longer therapeutic effect than inhibitors.

### DGlyTAC nbPDL1-PNGF for tumor suppression and immune enhancement

**Fig. 4:**
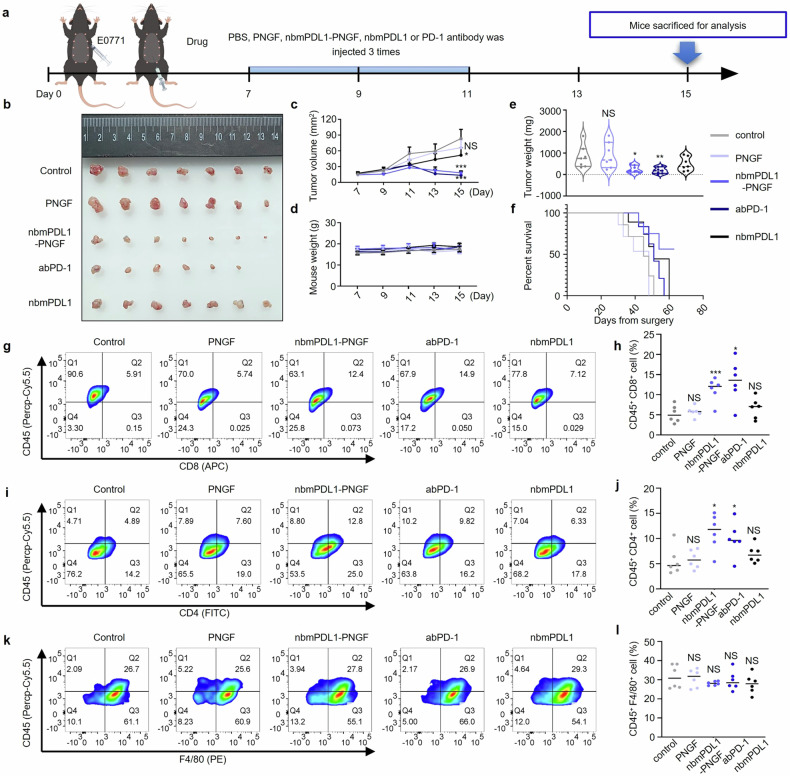
DGlyTAC nbmPDL1-PNGF prolonging survival of xenografted tumor mice and enhancing T-cell immunity in vivo. **a** Schematic illustration of the tumor-inhibition study and the general treatment procedure. E0771 cells were injected subcutaneously in the mammary pad of C57BL/6 mice. Drugs were administrated from day 7, at which point the tumors were ∼20 mm^2^ 5 mg/kg PNGF, nbmPDL1-PNGF, abPD-1 or nbmPDL1 was delivered via intraperitoneal injection every 2 days for 3 times, and PBS was used as a vehicle. **b** Comparison of dissected tumors after different treatments at day 15. **c** Tumor volume comparison at day 7, 9, 11, 13, 15. **d** Mouse weight comparison at day 7, 9, 11, 13, 15. **e** Tumor weight comparison of dissected tumors at day 15. **f** Survival curves for mice with different treatments (same as **a**). **g**, **I**, **k** Single-cell suspensions from E0771 tumors were stained for tumor-infiltrating CD4 and CD8 T cells as well as macrophage cells, and then determined by FACS. Subsets are depicted as percentage of all acquired live events; *n* ≥ 6 mice per group from five pooled, independent experiments. Large diagram: CD45 (Percp-Cy5.5), CD3 (BV510); smaller diagram: CD8 (APC), CD4 (FITC), CD8 (dark blue), CD11b (APC), F4/80 (PE). **h**, **j**, **l** The parameters analyzed were quantitated and are displayed as percentage of total CD4^+^ cells, CD8^+^ T cells, and macrophage cells, calculated from (**g**, **I**, **k**), respectively. (The box and error bars represent the mean ± SEM. *n* ≥ 6 mice per group, and a representative data was shown; ‘NS’ means not significant; ‘*’ means *p* < 0.05; ‘**’ means *p* < 0.01; ‘***’ means *p* < 0.001)

Since nbPDL1-PNGF inhibits the interaction between PD-L1 and PD-1 irreversibly and thus controls T cell activation, we then evaluated the nbmPDL1-PNGF antitumor activity in vivo. To confirm the prevention of nbmPDL1-PNGF in tumor growth, we engrafted C57BL/6 mice with E0771 cells to induce tumors. When the tumor size reached 4 × 5 mm^2^ PNGF, nbmPDL1-PNGF, mouse PD-1 antibody (abPD-1) or nbmPDL1 was delivered via intraperitoneal injection (Fig. 4a). Treatment of nbmPDL1 was a bit slower in tumor growth compared with PBS control; moreover, treatment with nbmPDL1-PNGF and abPD-1 markedly diminished tumor growth (Fig. 4b, c, e). While, the treatments had no effect on body weights (Fig. 4d, Supplementary Fig. 5e), suggesting there is no therapy-induced autoimmune disease. Next, we characterized the subgroups of intratumoral immune cells after treatment. As shown in Fig. 4g–l, a breakdown of CD45+ immune cells together with a detailed comparison of T cells were observed. Consistent with a potent immune activation and antitumorigenic activity of intratumoral T cells, we found evident immune deviation of CD8+ TILs and CD4+ TILs upon treatment of nbmPDL1-PNGF and abPD-1. Particularly in the subtype of E0771 triple-negative breast cancer, an increased immune infiltration was noted which represents better therapeutic responsiveness and improved survival chances (Fig. 4f).

Similar results were obtained in 4T1 (another triple-negative breast cancer cell line) tumor model, when PNGF, nbmPDL1-PNGF or nbmPDL1-PNGF-E206K was delivered via peri-tumoral injection every 3 days (Supplementary Fig. 5a). Mice with nbmPDL1-PNGF treatment underwent much slower tumor growth than that of control mice or nbmPDL1-PNGF-E206K treatment (Supplementary Fig. 5b–d). Same results were recapitulated in triple negative breast cancer in which 4th mammary glands were engrafted and evidenced by immunohistochemical staining with a monoclonal antibody against Ki-67 (Supplementary Fig. 5f, g). Thus, nbmPDL1-PNGF exhibited better therapeutic efficacy than PNGF and nbmPDL1-PNGF-E206K in immune-uncompromised mice model, suggesting a potential role of nbmPDL1-PNGF in cancer immunotherapeutics.

DGlyTAC nbmPDL1-PNGF shows much better effect than the inhibitor competitor nbmPDL1 in vivo, effectively inhibiting tumor growth and promoting the infiltration of immune cells. Indeed, nbmPDL1-PNGF has a similar efficacy to abPD-1, a better PD-L1/PD-1 interaction inhibitor than nbmPDL1. The frequent co-expression of PD-L2 on cells (like E0771 here) (Supplementary Fig. 4f) makes abPD-1 a better therapy over PD-L1 antibody (abPD-L1), since PD-L2 has much higher affinity for PD-1 than PD-L1.^39^

**Fig. 5:**
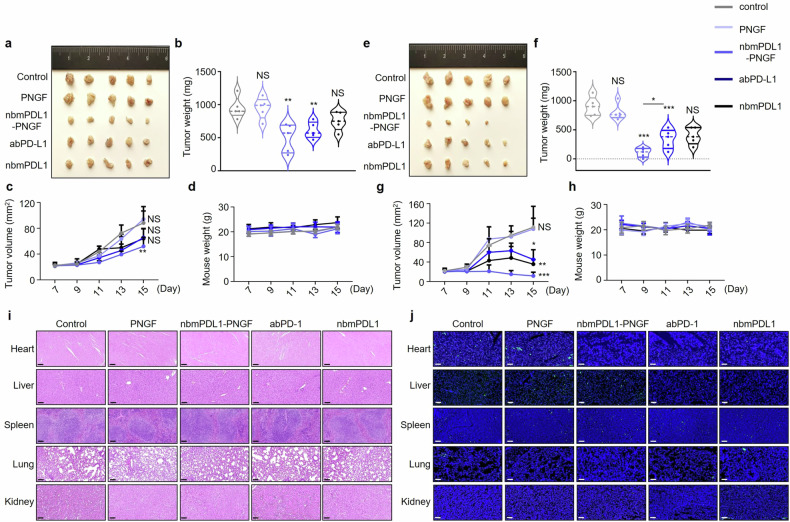
Anti-tumor efficacy and safety evaluation of DGlyTAC nbmPDL1-PNGF. MC38 cells were injected subcutaneously of C57BL/6 mice. Drugs were administrated from day 7, at which point the tumors were ∼20 mm^2^ 4 mg/kg (**a**–**d**) or 5 mg/kg (**e**–**h**) PNGF, nbmPDL1-PNGF, abPD-L1 or nbmPDL1 was delivered via intraperitoneal injection every 2 days for 3 times, and PBS was used as a vehicle. **a**, **e** Comparison of dissected tumors, (**b**, **f**) tumor weight, (**c**, **g**) tumor volume, and (**d**, **h**) mouse weight. **i** Representative H&E-stained and (**j**) TUNEL-stained sections of heart, liver, spleen, lung, and kidney from mice of different groups. The scale bars in the lower left corner of the image represent 100 μm. (The box and error bars represent the mean ± SEM. *n* ≥ 6 mice per group, and a representative data was shown; ‘NS’ means not significant; ‘*’ means *p* < 0.05; ‘**’ means *p* < 0.01; ‘***’ means *p* < 0.001)

MC38 cell line, a well-characterized model for colorectal cancer, was chosen to represent the therapeutic potential of DGlyTAC nbmPDL1-PNGF among various cancer types. MC38 had a minimal expression of PD-L2 (data not shown) to exhibit the best efficacy. We engrafted C57BL/6 mice with MC38 cells to form solid tumors. When the tumor size reached 4 × 5 mm^2^, 4 mg/kg (Fig. 5a–d) or 5 mg/kg (Fig. 5e–h) PNGF, nbmPDL1-PNGF, mouse abPD-L1 or nbmPDL1 was delivered via intraperitoneal injection 3 times. Inter-group comparison revealed an optimal effect of DGlyTAC nbmPDL1-PNGF in tumor growth inhibition (Fig. 5a–c, e–g). Indeed, DGlyTAC showed obvious improvement over abPD-L1 in 5 mg/kg dosage (Fig. 5f).

The glycosylation of PD-L1 can control its immunosuppressive function. In order to compare the inhibitory efficiency of an already marketed PD-1 inhibitor (Tislelizumab) and nbhPDL1-PNGF, we evaluated T cell responses by measuring apoptotic tumor cells co-cultured with primary human T cells (Supplementary Fig. 5h). In terms of enhancing the cytotoxicity of T cells, nbhPDL1-PNGF had better efficacy compared to Tislelizumab. In summary, the co-culture experiment of tumor cells and T cells provides us with an in vitro platform that simulates the in vivo environment, allowing us to compare the effects of different inhibitors and DGlyTACs on immunotherapy. We provide a comprehensive analysis that to understand the potential advantages or unique characteristics of nbhPDL1-PNGF.

### Tumor targeting and safety evaluation of DGlyTAC nbmPDL1-PNGF in vivo

PD-1 antibodies, well-established ICIs, are primarily used to treat various types of cancers. Although they effectively promote immuno-responses against cancer, immune reaction in normal tissues were also intrigued by PD-1 antibodies with associated organ toxicity.^40^ Similarly, we next clarified how the DGlyTAC nbmPDL1-PNGF was transported to different tissues, its retention in these tissues, and the toxicity in these tissues. Its distribution is crucial for evaluating the efficacy and safety of nbPDL1-PNGF as drugs in cancer treatment.

In the MC38 mouse model, both H&E and TUNEL stainings showed that nbmPDL1-PNGF did not change histologically in heart, liver, spleen, lung, and kidney tissues (Fig. 5i, j), accompanied by normal hematological parametric till the end of our study including hemoglobin levels plus different blood cell counts (Supplementary Fig. 6a). All these data showed no significant organ toxicity of DGlyTAC nbPDL1-PNGF, same as abPD-L1.

**Fig. 6:**
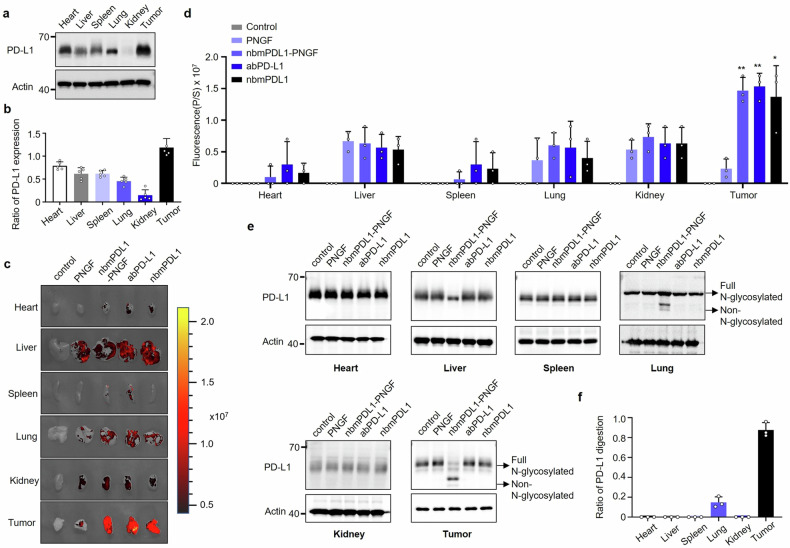
Biodistribution of DGlyTAC nbmPDL1-PNGF in vivo. **a** The expression of PD-L1 in various organs (heart, liver, spleen, lung, kidney, and tumor) isolated from C57BL/6 mice. **b** Quantitation of the PD-L1 level calculated from (**a**). **c** Mice were intraperitoneally injected with FITC labeled PNGF, nbmPDL1-PNGF, abPD-L1, or nbmPDL1, PBS as a vehicle (control). Representative IVIS images of peripheral tissues (heart, liver, spleen, lung, kidney, and tumor) at 12 h post-injection of FITC-labeled proteins. **d** Quantification of fluorescence signals in peripheral tissue (heart, liver, spleen, lung, kidney, and tumor) from (**c**). (The box and error bars represent the mean ± SEM. Independent experiment = 3, and a representative data was shown; ‘NS’ means not significant; ‘*’ means *p* < 0.05; ‘**’ means *p* < 0.01; ‘***’ means *p* < 0.001). **e** Representative immunoblotting of mPD-L1 in different organs and tumor of mice treated with PNGF, nbmPDL1-PNGF, abPD-L1, or nbmPDL1. Normally mPD-L1 has 4 ~ 5 N-glycans modified in vivo. For quantification of de-N-glycosylation efficiency, we regard mPD-L1 with 4 ~ 5 N-glycans modification as ‘Full-glycosylated’, and mPD-L1 with < 4 N-glycans modification as ‘Deglycosylated’. So the de-N-glycosylation efficiency is calculated as the ratio of the ‘Deglycosylated’ portion. **f** Quantification of PD-L1 de-N-glycosylation efficiency calculated from (**e**). (The box and error bars represent the mean ± SEM. *n* ≥ 5 mice per group, and a representative data was shown; ‘*’ means *p* < 0.05; ‘**’ means *p* < 0.01)

Then, we examined the expression of PD-L1 in various organs (heart, liver, spleen, lung, kidney and tumor) isolated from MC38-bearing mice. Heart, liver, spleen, lung and tumor except kidney have certain amounts of PD-L1 expression (Fig. 6a, b, Supplementary Fig. 7a–d). To verify the clinical application potential of nbmPDL1-PNGF, its bio-distribution was tested by IVIS imaging system in animal model. FITC labeled PNGF, nbmPDL1-PNGF, abPD-L1 or nbmPDL1 was intraperitoneal injected into the MC38-bearing mice. About 40%–60% of the injected dose could be detected in tumors that are highly expressing PD-L1 (Fig. 6c, d, Supplementary Fig. 7e). We also found that nbmPDL1-PNGF, abPD-L1 and nbmPDL1 abundantly distributed in the liver, lung and kidney (Fig. 6c, d, Supplementary Fig. 7e). In addition, very few de-N-glycosylation was observed in the heart, spleen and kidney; and although nbmPDL1-PNGF were distributed in various organs (liver, lung and tumor), the highest and nearly complete de-N-glycosylation efficiency of nbmPDL1-PNGF was detected in tumors (Fig. 6e, f, Supplementary Fig. 6b–g). Only lung exhibited a bit de-N-glycosylation with DGlyTAC nbmPDL1-PNGF, so we may predict the highest clinical toxicity will come from lung; and much lower toxicity towards liver and nearly none for other organs. While, abPD-L1 and abPD-1 both show high clinical toxicity toward lung, liver and kidney.^40^ The accumulation of DGlyTAC in the heart and spleen is minimal. Although DGlyTAC is enriched in the liver and kidneys, no significant de-N-glycosylation was observed (Fig. 6e). Furthermore, the expression levels and glycosylation states of PD-L1 in these organs are relatively low. As a result, the enrichment of DGlyTAC in the liver and kidneys may not be solely due to its binding with PD-L1, but could also stem from conventional protein metabolic processes.

In this study, we evaluated the potential of non-pathogenic nbPDL1-PNGF for tumor-targeting therapy. Our data showed that it could colonize solid tumors efficiently. Moreover, this is the first attempt to use DGlyTAC nbPDL1-PNGF as a tumor-targeting N-glycosidase to significantly restrain the growth of tumors. We conclude that DGlyTAC nbPDL1-PNGF can be a better therapy than abPD-L1 (Fig. 5) while maybe with lower toxicity (Fig. 6).

## Discussion

**Fig. 7:**
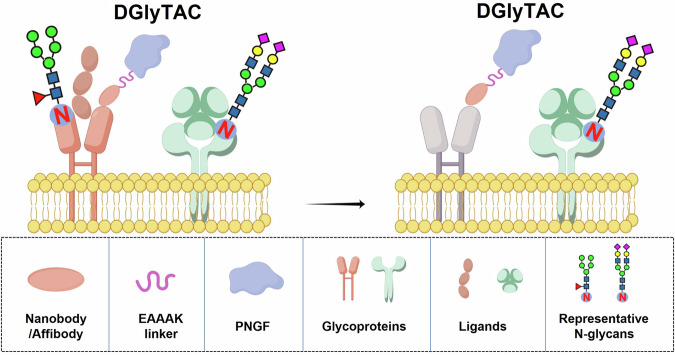
Design and development of a DGlyTAC to remove N-glycans in a protein-specific manner. The Nb/Af is able to recognize target protein and let PNGF to erase the N-glycans. The enhanced de-glycosylation effect was achieved by the recruitment of PNGF with an N-terminal Nb/Af against target protein. In the meanwhile, a stiff α-helical linker with the sequence of A(EAAAK)nA (*n* = 4) was introduced between Nb/Af and PNGF. The design is guided by in situ N-glycan removal of target proteins without affecting other N-glycoproteins, and is used to realize the research of biological functions and related molecular mechanisms based on N-glycans. The target proteins used in this study were eGFP-CD24, CD47, IGF1R, PD-L1, EGFR and HER2. The corresponding Nb/Afs were nbGFP, nbCD47, afIGF1R, nbPD-L1, afEGFR and afHER2. (FigureDraw ID: STITY8cf46)

We have indicated the generality of DGlyTACs using four nanobodies and three affibodies directed against seven protein targets representing a wide range of N-glycosylated substrates. Our project creates a universal strategy for enzyme engineering to specifically remove the N-glycans of glycoproteins on the surface of living cells (Fig. 7, FigureDraw ID: STITY8cf46). We have chosen PNGF from Flavobacterium meningosepticum, which is an enzyme widely used in protein glycosylation research. It can specifically hydrolyze N-linked oligosaccharides on glycoproteins or glycopeptides, thereby releasing intact N-glycan chains.^16^ When DGlyTACs are applied alternately to the target proteins, they do not interfere with the N-glycosylation modifications of non-target proteins, showing perfect orthogonality (Supplementary Fig. 8b-c). So this design is guided by in situ N-glycan removal of target proteins while minimally affecting other cell surface N-glycosylated proteins. The DGlyTACs enable the specific de-N-glycosylation of a protein of interest in cells and complements chemical and genetic methods to perturb N-glycosylated levels of targets. DGlyTACs are tailored for the targeted inactivation of membrane proteins through the specific erasing of N-glycans. Finally, we applied the system to characterize the importance of N-glycans to target proteins in the context of cellular biological functions and tumor immunotherapy. PNGF does possess immunogenicity in humans or mice. However, mice do not exhibit acute immune responses.

It is well-known that PROTACs are designed to selectively degrade target proteins by exploiting the ubiquitin-proteasome pathway within cells. Upon entering a cell, PROTAC molecules function as molecular bridges that bring the target protein into close proximity with an E3 ubiquitin ligase. This interaction facilitates the ubiquitination of the target protein, marking it for recognition and subsequent degradation by the proteasome.^41^ For this mechanism to be effective, PROTACs must be able to traverse the cell membrane; thus, their design must prioritize optimizing cell permeability to ensure efficient intracellular access and functional activity. LYTAC technology, an innovative approach for targeted protein degradation, exhibits considerable potential for the specific degradation of extracellular and membrane proteins. This method operates through the engagement of cell surface lysosomal targeting receptors (LTRs), such as CI-M6PR or ASGPR, which facilitate the endocytosis and subsequent lysosomal degradation of the target proteins.^42^ Consequently, the efficacy of LYTACs is inherently dependent on the expression levels and distribution of these receptors, which can limit their overall effectiveness and applicability. DGlyTACs share conceptual parallels with targeted protein degradation strategies such as PROTACs and LYTACs, yet it operates independently the presence of degradation-associated proteins (such as E3 ligases and ASGPR), sharing the same targets and clinical indications as antibody inhibitors. Similar to PROTACs and LYTACs, the DGlyTACs works in an irreversible and recyclable manner, enabling protein inactivation at a lower concentration over inhibitors. Moreover, DGlyTACs are of rapid action (at least as less as 2 h) and long effect (~24 h even after DGlyTACs were swept out), making it a good candidate as the next generation antibody-related drugs. The maximal recycling capacity of DGlyTACs is the same as the total turnover number of the enzyme PNGF, which could be enhanced using enzymatic directed evolution.

The DGlyTACs we designed and the antibody-sialidase conjugates previously developed by Carolyn R. Bertozzi can both be utilized for cancer immunotherapy.^43^ Nonetheless, there exist distinct disparities in their practical applications. Primarily, their drug targets are different: DGlyTACs engage in protein-specific deglycosylation (wherein the target protein constitutes the drug target), whereas the antibody-sialidase conjugates execute cell-specific desialylation (with sialic acids serving as the drug target). Secondly, the efficacy of the antibody-sialidase conjugates necessitates a sufficiently high expression level of the target protein; conversely, DGlyTACs merely require that the N-glycosylation of target proteins possesses biological functionality. Additionally, their construction methodologies are different: DGlyTACs incorporate a fixed linker, ensuring that, upon binding of the nanobody to the target protein, the distance between the target protein and the enzyme approximates 4 nm, thereby enabling PNGF to act exclusively on the target protein. In contrast, within the antibody-sialidase conjugates, the distance between the target protein and the enzyme remains indeterminate (e.g., 4–10 nm), permitting the sialidases to affect not only the target protein but also neighboring proteins. Furthermore, to effectuate the removal of sialic acid modifications across the entire cell, the antibody-sialidase conjugates demands a higher concentration for its operation (specifically exceeding 10 nM); whereas low concentration of DGlyTACs (no more than 5 nM) exhibits high efficacy alongside low toxicity.

The advantages of protein therapeutics lie in their high specificity and biological activity, enabling precise modulation and intervention in specific biological processes. They have a broad therapeutic range, applicable to various diseases, and possess the potential to quickly respond to clinical needs.^44^ As a promising protein therapeutic, DGlyTACs achieve protein inactivation by removing N-glycans from target proteins in situ. Because not all N-glycosylation modifications exhibit clearly defined biological functions,^45^ the comprehensive functional study of these N-glycosylation is of great urgency before applying DGlyTACs to a series of target proteins and subsequent clinical trials. Fortunately, DGlyTAC technique itself is an in situ tool to study the functions of N-glycans on target proteins, facilitating the research and therapeutic application at the same time. In future, large numbers of DGlyTACs will be made for specific studies on the function of N-glycans and subsequent potential therapeutics, creating a new era for glycobiology and glycoproteins as drug targets.

In summary, we have developed a method using Nb/Af to target and inactivate extracellular and membrane-associated glycoproteins, which we refer to as DGlyTAC. The DGlyTAC platform can directly target glycoproteins, promoting the removal of N-glycans, thereby effectively inactivating the target proteins. We have showed that DGlyTACs exhibit good target specificity and potent immunotherapeutic capacity with ICs as targets. Like antibody drugs, the off-target organ toxicity will be the greatest challenge for further translational applications for the non-specific expression of target proteins on tumors. We anticipate that the tunability and modularity of DGlyTACs will provide new opportunities for protein drug designs and maybe avoiding some side effects, enabling target protein de-N-glycosylation for both research and potential therapeutic applications.

## Materials and methods

### Ethic statements

Mice were obtained from GemPharmatech and bred in the animal facility of the Central Laboratory of Tongji University Affiliated Tenth People’s Hospital. Animal handling and experimental procedures were approved by the Institutional Animal Care and Use Committee at Tongji University. The approval number for the animal experiments in this study is ‌SHDSYY-2024-7005‌.

### Cell culture

The human cancer MCF7, MDA-MB231, SK-BR-3, PANC1, HepG2, A375, SW620 cells, and mouse cancer E0771 and 4T1 cells were a gift from the lab of Professor Tingxiu Xiang in Chongqing University Cancer Hospital. The CHO cell line was a gift from the lab of Professor Xing Chen at Peking University. MCF7, MDA-MB231, SK-BR-3, PANC1, HepG2, E0771, and 4T1 cells were maintained in RPMI 1640 medium (BasalMedia) supplemented with 10% FBS (ExCell). SW620, HeLa, and A375 cells maintained in DMEM medium (BasalMedia) supplemented with 10% FBS (ExCell) and were cultured in a humidified incubator at 37 °C under 5% CO_2_. The CHO-CD24 cells stably expressing eGFP-CD24 were constructed in a pcDNA3.1 vector containing the eGFP-CD24 gene, followed by sorting based on eGFP fluorescence.

### Tumor challenge

C57BL/6 mice were implanted subcutaneously with 1 × 10^6^ E0771, or 5 × 10^5^ MC38 cells to induce tumors. When the tumor size reached 4 × 5 mm^2^, evaluation of the antitumor efficacy of 4 mg/kg or 5 mg/kg PNGF, nbmPDL1-PNGF, abPD-1 or nbmPDL1 was delivered via intraperitoneally every 2 days for 3 times, and PBS was used as a vehicle. We measured the diameters of the perpendicular tumors with calipers and calculated the tumor volume using the formula below: Tumor area (mm^2^) = d*D, where D and *d* are the longest and shortest diameters of the tumor (in millimeters), respectively. Mice (for tumor size comparison and immune cell infiltration assessment) were sacrificed 15 days after tumor challenge. The sacrifice criteria for mice undergoing survival analysis (60 days): tumor area (mm^2^) X3.14/4 > 200 mm.^2^

2 × 10^5^ 4T1 cells were injected subcutaneously in the mammary pad of balb/c mice. Drugs were administrated from day 6, at which point the tumors were ∼100 mm^3^. Evaluation of the antitumor efficacy of 2 mg/kg PNGF, nbmPDL1-PNGF or nbmPDL1-PNGF-E206K was delivered via peri-tumoral injection every 3 days, and PBS was used as a vehicle. Mice were sacrificed 30 days after tumor challenge. Tumor volumes were measured in two dimensions (length and width) and the volume was calculated using the formula, tumor size (mm^3^) = (length × width^2^) × 0.5.

### Antibodies and reagents

The primary antibodies for eGFP (Santa Cruz Biotechnology, Cat# sc-9996), IGF1R (Signalway Antibody, Cat# 21081), PD-L1 (Cell Signaling Technologies, Cat# 29122; Abcam, Cat# EPR20529), HER2 (Cell Signaling Technologies, Cat# 2165), EGFR (Cell Signaling Technologies, Cat# 2646), InVivoMab anti-mouse PD-1/PDL1 (Bioxcell, Cat# BE0146-50MG/BE0101-5MG), and Actin (Proteintech, Cat# 66009-1-lg), H2-K1 (Abnova, Cat# PAB16017). The secondary antibodies were goat anti-rabbit IgG, HRP-linked (Abcam, Cat# ab205718), goat anti-mouse IgG, HRP-linked (Abcam, Cat# ab205719), streptavidin (HRP) (Abcam, Cat# ab7403), Cy5-Streptavidin (Invitrogen, Cat# 434316), and biotin labeled antibody goat anti-human IgG Fc (Thermo Fisher Scientific, Cat# A18821), ConA-Biotin (Xi’an Qiyue Biology, Cat# R-FC-005). Recombinant proteins human PD-1 Fc chimera (Cat# 1086-PD-050) and human Siglec-10 Fc chimera (Cat# 2130-SL-050) were purchased from R&D Systems.

### Western blotting

For electrophoresis, 40 µg of protein samples were loaded into 10% or 12% sodium dodecyl sulfate-polyacrylamide gel (SDS-PAGE), and transferred onto the 0.22 μm/0.45 μm polyvinylidene fluoride (PVDF) membrane (Millipore). After protein transfer, the membranes were blocked in 5% (w/v) skimmed milk in PBST buffer for 2 h at room temperature with gentle shaking. We incubated the membranes overnight at 4 °C with a primary antibody solution (5% BSA or 5% PBST). After incubation with primary antibodies, the membranes were washed with PBST buffer for 3 times at room temperature with gentle shaking. The membranes were then incubated with HRP-conjugated goat anti-rabbit and anti-mouse IgG antibodies (1:30000 dilution) for 1 h at room temperature. Finally, the membranes were washed again with PBST buffer for 3 times (each for 10 min) at room temperature with gentle shaking. Western blot bands were detected with a substrate that emits electro-chemiluminescence (ECL) (ShareBio, Cat# SB-WB012).

### Immunoprecipitation (IP)

An IP assay was carried out to investigate the interaction between glycosylation and the target protein. Briefly, total proteins of the CHO-CD24 cells were extracted with ice-cold low-salt lysis buffer, and then the cell lysate was incubated with the eGFP antibody overnight on a turning wheel at 4 °C. The MilliporeSigma Protein A/G Mix Magnetic Bead System (Cat#LSKAGAG10) was used to purify the co-IP complex, and the low salt lysis buffer was used to wash the beads. Then the co-IP complex was separated into two samples, one for eGFP-CD24 western blot and the other for ConA -based streptavidin blotting.

For PD-L1, total proteins of the MDA-MB231 were extracted with ice-cold low-salt lysis buffer, and then the cell lysate was incubated with the PD-L1 antibody overnight on a turning wheel at 4 °C. The MilliporeSigma Protein A/G Mix Magnetic Bead System (Cat#LSKAGAG10) was used to purify the co-IP complex, and the low salt lysis buffer was used to wash the beads. Then the co-IP complex was separated into two samples, one for PD-L1 western blot and the other for Fucosyltransferase-based streptavidin blotting.

### Enrichment of Glycopeptides

We transfected pcDNA3.1-eGFP-CD24 or pcCDH-H2-K1 plasmid into CHO cells (termed CHO-CD24 or CHO-H2-K1) which lack of CD24 or H2-K1expression. After co-incubation of cells with enzymes at 37 °C for 2 h, cells were dissolved in lysis buffer at a fivefold volume (4% SDS), followed by ultrasonication for 5 min and centrifugation at 12,000 g and 18 °C for 30 min to obtain the protein extracts. Subsequently, six volumes of acetone were added to precipitate the proteins at −20 °C overnight. The precipitates were redissolved after 5 minutes of ultrasonication, and then diluted tenfold with 100 mM NH_4_HCO_3_. The concentration of proteins was measured using the BCA method. Next, the proteins were reduced in 10 mM TCEP at 57 °C for 30 min, and subsequently alkylated in the dark with 20 mM IAA at room temperature for 30 minutes. After that, the proteins were digested into peptides by incubating them with trypsin at a final enzyme - to - substrate ratio of 1:50 at 37 °C overnight. The resulting peptides were desalted using Sep - Pak C18 cartridges (Waters, USA). Finally, the desalted peptides were dried by vacuum centrifugation and then employed for glycopeptide enrichment. Glycopeptides were enriched by zwitterionic hydrophilic interaction liquid chromatography (ZIC-HILIC) method. Briefly, the desalted peptides of 1 mg were resuspended in 300 μL loading buffer containing 80% ACN and 1% TFA and then loaded onto a homemade micro-column containing 50 mg of ZIC-HILIC particles (Merck Millipore, Darmstadt, Germany) packed onto a C8 disk. The flow-through was collected and reloaded onto the column for additional four times. Then, the column was washed with 200 μL loading buffer for four times, and finally eluted with 140 μL 0.1% TFA. The elution was collected and lyophilized.

### LC-MS/MS for glycopeptide analysis

Glycopeptide analysis was conducted using an Orbitrap Fusion Tribrid mass spectrometer (Thermo Scientific) in conjunction with an EASY-nano-LC system (Thermo Scientific) without the trap column. Samples were loaded onto a C18 spray tip column (50 cm × 75 µm i.d.) and separated at a flow rate of 300 nL/min. The mobile phase consisted of Solvent A (0.1% formic acid in water) and Solvent B (acetonitrile with 0.1% formic acid), with a gradient elution profile: 5% B increasing to 35% B over 345 min, followed by a 5-minute ramp to 90% B, and holding at 90% B for an additional 10 min.

For glycopeptide analysis, the MS parameters were set as follows: scan range from 800 to 2000 m/z, resolution of 120,000, AGC target of 200,000, maximum injection time of 100 ms, and inclusion of charge states from 2 to 6. Dynamic exclusion was enabled after one occurrence, with an exclusion duration of 15 s. Each selected precursor ion was subjected to one HCD-MS/MS event.

The HCD-MS/MS settings included an isolation window of 4 m/z, detection by the Orbitrap, resolution of 15,000, AGC target of 500,000, maximum injection time of 250 ms, and collision energy of 30%. Stepped collision mode was activated with an energy difference of +10%, where 10% represents the absolute value in the Orbitrap Fusion.

### ConA-based streptavidin blotting

In this study, we employed a straightforward method for detecting glycans with ConA-based streptavidin blotting. Concanavalin A (ConA) is a lectin that specifically binds to α-D-mannose and α-D-glucose residues. By conjugating ConA with biotin, it can be detected using streptavidin-conjugated enzymes, which allows for sensitive and specific detection of mannose in samples. Add 2 μg/mL of ConA-biotin to the cell sample. Incubate at room temperature for 1−2 h to allow ConA-biotin to bind to mannose residues. Remove unbound ConA-biotin by washing the plate with PBS for 3 times. The protein samples were separated by SDS-PAGE and then transferred onto a nitrocellulose membrane, and incubated for another 30 min to allow binding of the streptavidin-HRP to the biotinylated ConA. Finally, the membranes were washed again with PBST buffer for 3 times (each for 10 min) at room temperature with gentle shaking. Western blot bands were detected with a substrate that emits electro-chemiluminescence (ECL).

### Fucosyltransferase-based Chemoenzymatic Glycan Labeling

For enrichment and identification of N-glycosylation proteins, cells were lysed in RIPA buffer and re-suspended in buffer containing 1% SDS at a concentration of 1 mg/mL in a six-well plate. 50 µl suspensions obtained above were incubated with 50 μM CuSO_4_-BTTAA premixed complex (CuSO_4_-BTTAA, molar ratio 1:2), 100 μM alkyne-Biotin, 100 μM UDP-FucAz, 40 μM FucT, and 2.5 mM fresh sodium ascorbate for click reaction at 37 °C for 30 min. Remove unbound mixture by washing the plate with PBS for 3 times. The protein samples were separated by SDS-PAGE and then transferred onto a nitrocellulose membrane, and incubated with streptavidin-HRP for another 30 min. Finally, the membranes were washed again with PBST buffer for 3 times (each for 10 min) at room temperature with gentle shaking. Western blot bands were detected with a substrate that emits electro-chemiluminescence (ECL).

### Removal and detection of cellular sialic acid

Replate CHO-CD24 cells onto a 12-well plate, add concentration gradients P-3Fax-Neu5ac (50 μM, 100 μM, 200 μM, and 400 μM) for 24 h, then add Ac4ManNAz (200 μM/well), and treat for another 48 h. Transfer cells to 1.5 ml EP tubes, and wash with PBS twice. 50 µl suspensions obtained above were incubated with 50 μM CuSO4-BTTAA premixed complex (CuSO_4_-BTTAA, molar ratio 1:2), 100 μM alkyne-Biotin, 100 μM UDP-FucAz, 40 μM FucT, and 2.5 mM fresh sodium ascorbate for click reaction at 37 °C for 30 min. After being incubated with cy5-streptavidin for another 30 min, the cellular sialic acid was analyzed by flow cytometry.

### Plasmid construction

The expression plasmid pCDNA3.1-eGFP-CD24 was constructed by cloning the fused fragment of signal peptide of CD24, eGFP, and encoding CD24 sequence into the pcDNA3.1 vector.

The expression plasmid pET21a-NbGFP-PNGF was constructed by fusing eGFP nanobody, linker, and PNGF. The plasmids pET28a-PNGF, pET21a-NbGFP-PNGF, pET21a-NbCD47-PNGF, pET28a-NbhPdL1-PNGF, pET28a-NbmPdL1-PNGF, pET28a-NbmPdL1, pET28a-AfIGF1R-PNGF, pET21a-AfEGFR-PNGF and pET21a-AfHER2-PNGF were constructed in a similar way. All constructed protein sequences were codon optimized for expression in *Escherichia coli*, synthesized in Sangon Bioengineering (Shanghai), and inserted into pET21a / pET28a plasmid using seamless ligation (between XbaI site and 6xHis-Tag). These plasmid sequences were listed in Supplementary ‘DNA sequence of plasmids’.

In parallel, the plasmids for the enzyme PNGF, which is completely inactive, were generated by mutating PNGF at catalytic residue 206 from glutamic acid to lysine, and the corresponding primers were used to perform site-directed mutagenesis on each vector in order to obtain pET21a-NbGFP-PNGF-E206K, pET21a-NbCD47-PNGF-E206K, pET28a-NbhPdL1-PNGF-E206K and pET28a-NbmPdL1-PNGF-E206K.

### Sequences of nanobodies and affibodies

NbGFP: VQLVESGGALVQPGGSLRLSCAASGFPVNRYSMRWYRQAPGKEREWVAGMSSAGDRSSYEDSVKGRFTISRDDARNTVYLQMNSLKPEDTAVYYCNVNVGFEYWGQGTQVTVSS

AfIGF1R: VDNKFNKEGFYAAIEILALPNLNRKQSTAFISSLEDDPSQSANLLAEAKKLNDAQAPK

NbhPDL1: QVQLQESGGGLVQPGGSLRLSCAASGKMSSRRCMAWFRQAPGKERERVAKLLTTSGSTYLADSVK

NbmPDL1:

GRFTISQNNAKSTVYLQMNSLKPEDTAMYYCAADSFEDPTCTLVTSSGAFQYWGQGTQVTVSS

AfHER2: VDNKFNKEMRNAYWEIALLPNLNNQQKRAFIRSLYDDPSQSANLLAEAKKLNDAQAPK

AfEGFR: VDNKFNKEMWAAWEEIRNLPNLNGWQMTAFIASLVDDPSQSANLLAEAKKLNDAQAPK

NbCD47:

EEELQIIQPDKSVLVAAGETATLRCTITSLFPVGPIQWFRGAGPGRVLIYNQRQGPFPRVTTVSDTTKRNNMDFSIRIGNITPADAGTYYCIKFRKGSPDDVEFKSGAGTELSVRAKP

### Expression and purification of proteins

Plasmid pET21a-NbGFP-PNGF was transformed into BL21(DE3) competent cells. For expression of NbGFP-PNGF, transformed bacteria were cultured at 37 °C in 2×TB media with 100 μg/mL ampicillin and induced with 1 mM IPTG at 20 °C when OD600 reached 0.8 ~ 1.2. After induction of expression at 20 °C for 24 h, bacteria were harvested by centrifugation at 8000 rpm for 5 min at 4 °C. After re-suspending the pellets in PBS buffer five mL per gram of wet weight, the cell suspension was sonicated for 20 minutes in an ice-water bath. After the centrifugation at 20,000 g for 30 min at 4 °C, the supernatant was loaded into a Ni-NTA column. The columns were washed with wash buffer (20 mM Tris, pH 8.0, 150 mM NaCl, and 20 mM imidazole) for 10 columns. The protein was then eluted with elution buffer (20 mM Tris, pH 8.0, 150 mM NaCl, and 500 mM imidazole). Purified proteins were then dialyzed into PBS at 4 °C for 3 times (each for 4 h). Other proteins, NbGFP-PNGF-E206K, NbCD47-PNGF, NbCD47-PNGF-E206K, NbhPDL1-PNGF, NbhPDL1-PNGF-E206K, NbmPDL1-PNGF, NbmPDL1-PNGF-E206K, PNGF, AfIGF1R-PNGF, AfEGFR-PNGF, AfHER2-PNGF and NbmPDL1 were expressed and purified in a similar way. In Supplementary Fig. 8a, SDS-PAGE result indicating the production and purity of all the recombinant proteins.

### Immune receptor and ligand interaction assay

To measure PD-1 and PD-L1 protein interaction, we treated MDA-MB231 with 5 nM PNGF, nbhPDL1-PNGF, and nbhPDL1-PNGF-E206K in a humidified incubator at 37 °C under 5% CO_2_ for 24 h. After washing cells with PBS buffer for 30 minutes at room temperature with gentle shaking, we incubated them with recombinant human PD-1 Fc chimera for 2 hours. The biotin labeled antibody goat anti-human IgG Fc was used at 4 °C overnight. The secondary antibody used here was anti-biotin Cy5 dye conjugate streptavidin. Flow cytometry measured PD-1 binding on the membrane of MDA-MB231 cells expressing PD-L1. We used a similar method to measure the interaction between CD24 and Siglec-10. Before the cells were incubated with recombinant human Siglec-10 Fc chimera, the bound PNGF, nbGFP-PNGF, and nbGFP-PNGF-E206K were dissociated in acidic buffer (100 mM Glycine, 150 mM NaCl, pH 3.0 containing 0.1% Tween-20) for 2 min.

### PD-L1, PD-1, and nbhPD-L1 interactions alignment

Upon alignment in PyMOL, the structures of PDB 4zqk (binding of PD-L1 to PD-1) and PDB 5jds (binding of PD-L1 to nbhPD-L1) are obtained. PD-L1 is depicted in white and green, PD-1 in pink, and nbhPD-L1 in blue. The findings indicate that PD-1 and nbhPD-L1 utilize the same binding site on PD-L1.

### Isolation of macrophages from bone marrow

Eight-week-old C57BL/6 wild-type mice were acquired from Shanghai Slaccas Laboratory Animal Company Limited. In order to obtain bone marrow-derived macrophages (BMDM), femurs and tibias were perfused with ice-cold phosphate - buffered saline (PBS), and red blood cells were lysed by red blood cell lysis buffer. Subsequently, bone marrow cells were cultivated in Dulbecco’s modified Eagle’s medium (DMEM) supplemented with 10% fetal bovine serum (FBS), 100 U/mL penicillin, and 100 μg/mL streptomycin in the presence of 50 ng/mL murine macrophage-colony stimulating factor (M-CSF) (PeproTech), 0.1 mM sodium pyruvate (Gibco, Cat# 11360-70) and 10 mM N-2-hydroxyethylpiperazine-N’-2-ethanesulfonic acid (HEPES) (Gibco, Cat# 15630-080) for seven days.

### Phagocytosis assay

Macrophages were plated 1 × 10^5 cells per well in a 48-well plate in complete DMEM medium, cancer cells were stained with Calcein, AM (Yeasen, China) at 37 °C for 10 min. Each phagocytosis reaction reported in this work was performed by co-culture of target cells and macrophages for 4 h at 37 °C. Macrophages were identified with APC-labeled anti-F4/80 (Biolegend, Cat. # 123116), and flow cytometry (BD FACSCanto II, San Jose, CA) was performed. Cytochalasin D were purchase from MCE (HY-N6682). After the successful induction of mouse macrophages, they were treated with a concentration of 10 μM for 1 hour to inhibit cytoskeleton remodeling, and then CHO-CD24 cells were added to the cell culture dishes containing or without Cytochalasin D for phagocytosis experiments. Use flow cytometry to analyze cell phagocytic activity and observe the proportion of double-positive cells. Phagocytosis was calculated as the percentage of Calcein+F4/80+ cells (Q2) among Calcein+ cells (Q2 + Q3): phagocytosis (%) = [Q2 / (Q2 + Q3)] x100%.

### Kd determination using isothermal titration calorimetry

In Kd screening, antibody or ligands were loaded into sensors using 20 nM solutions. A baseline was established in PBS containing 1 mg/ml BSA, followed by submersion of sensors in a single concentration of analyte in the assay buffer. The dissociation was monitored in fresh assay buffer during the dissociation process. A sensor shake rate of 1000 rotations per minute was used throughout all experiments. Using ForteBio’s data analysis software (Menlo Park, CA, USA), a 1:1 binding model was fitted to the data in order to calculate the association rate and dissociation rate. The ratio of kd : ka was used to calculate Kd. The antigen NbGFP-PNGF (10 nM) was pre-incubated at room temperature for one hour with a second antibody (10 nM) in a typical epitope binning assay. An AMC sensor (ForteBio) was loaded with a control antibody (20 nM). A mixture of second antibodies and antigens was pre-incubated on sensors. ForeBio Data Analysis Software 7.0 was used to process raw data and assess competitive binding between antibody pairs. Antibodies binding to additional epitopes indicate unoccupied epitopes (non-competitors), and no binding indicates epitope blocking (competitors).

### Immunohistochemistry (IHC)

In brief, paraffin sections were dewaxed, rinsed twice with absolute ethanol, and hydrated, and antigens were extracted according to the instructions (ZSGB-BIO, SP9000). After incubating for 10 min at room temperature with reagent 1, the slides were washed three times in PBS for 3 min each. Then sections were incubated in blocking solution (reagent 2) for 15 minutes, followed by overnight incubation at 4 °C with ki-67 antibody in an immunohistochemical wet box. The following day, sections were washed three times with PBS, incubated with reagent 3 at 37 °C for 30 min in a wet box, washed again with PBS, and then incubated with reagent 4 at 37 °C for 30 min. After being washed three times with PBS, immunolabeling was performed with 1x diaminobenzidine (DAB, ZSGB-BIO, Cat# ZLI-9018). Finally, the sections were counterstained with hematoxylin (Biosharp, China, Cat# BL702B).

### Immunofuorescence staining

E0771 cell was incubated with whole goat serum containing primary antibody against PD-L1 (Abcam, Cat# EPR20529) overnight at 4 °C. Then, the cells were incubated with biotin-labeled secondary antibody (Jackson ImmunoResearch, Cat# 111-065-003) for an additional 20–30 min. At the same time, nuclei were counterstained with DAPI (Beyotime, Cat# C1341M). The staining was observed under fuorescence microscopy.

### Hematoxylin and Eosin (H&E) staining

An organ tissue sample from a mouse was fixed in 4% paraformaldehyde for 12 h, then embedded in paraffin, cut into 4 µm slices, and mounted on a microscope slide. Following slide placement in a slide holder, two changes of xylene were used to dissolve paraffin, followed by sequential changes of 100, 95, and 70% ethanol for rehydration. Hematoxylin was applied to the slides and incubated at room temperature for eight minutes to stain the nuclei. Eosin staining was performed for 30 s after tissue samples were thoroughly washed with tap water and differentiated with 1% acid ethanol. A light microscope was used to acquire images of tissue samples.

### Immunofluorescence histochemical analysis

An organ tissue sample from a mouse was fixed in 4% paraformaldehyde for 12 h, then embedded in paraffin, cut into 4 µm slices, and mounted on a microscope slide. TUNEL staining was applied to the paraffin-embedded tissue sections. Fluorescence images of each tissue section were obtained and analyzed by ImageJ.

### Tumor digestion and mAbs

On harvest day, mice were euthanized, and tumors were mechanically dissociated and digested with deoxyribonuclease (10000 Kunitz/mL, Sigma, Cat# DN25/DN4527) and collagenase IV (Thermo, Cat# 17104019). After lysing red blood cells with lysing buffer, tumor cells were resuspended in FACS buffer. Tumor digests were filtered and resuspended as single cell suspensions. Cells were treated with Fc block (BD Pharmingen, Cat# 564219), then stained with Amcyan live/dead dye (Thermo Scientific Fisher, Cat# L10119). In order to analyze with flow cytometry, single-cell suspensions were prepared and stained with the indicated markers. For antibody staining, cells were washed with PBS containing 2% FBS, and stained on ice.

The antibodies used were CD45-Percp-Cy5.5 (BioLegend, Cat#103132), CD3-BV510 (BioLegend, Cat#100233), CD8-APC (BioLegend, Cat#100712), and CD4-APC (BioLegend, Cat#100553), CD11b-APC (BioLegend, Cat# 301310), F4/80-PE (BioLegend, Cat# 123109). Dead cells were excluded using the near-infrared dye (Invitrogen, Cat# 65-0865-14). Flow analysis was performed using BD FACS Canto II (San Jose, CA), and singlets were gated on using FSC-H versus FSC-A.

### The biodistribution study

The biodistribution of antibodies was performed on C57BL/6 mice subcutaneously bearing MC38 tumors. The mice were intravenously injected with FITC-labeled PNGF, nbmPDL1-PNGF, abPD-L1, and nbmPDL1 (200 μl). The mice were sacrificed after 12 h, and their major organs were collected, including the heart, liver, spleen, lung, kidney, and tumor. In vivo imaging was performed using an in vivo imaging system (IVIS) under the same settings. The fluorescence intensity was measured using Living Image software.

### T cell-mediated tumor cell killing assay

The T cell-mediated tumor cell cytotoxicity assay was conducted in accordance with the adapted protocol provided by Essen Bioscience. In summary, to activate tumor-specific T cells, we incubated tumor cells together with anti-CD3 antibodies (proteintech, Cat# 65133-1-Ig) and interleukin-2 (IL-2) (MedChemExpress, Cat# HY-P7037B)-activated human peripheral blood mononuclear cells for a duration of 5 to 7 days. Subsequently, we isolated and expanded the T cell population employing the ImmunoCult Human CD3/CD28 T Cell Activator (Cell Signaling Technologies, Cat #70976). To analyze the killing of tumor cells by T cell, we co-cultured tumor cells which treated with different deglycosylases or Tislelizumab (MedChemExpress, Cat# HY-P99052). T cells, specifically cytotoxic T lymphocytes, identify and bind to tumor cells, subsequently releasing perforin and granzymes. These cytotoxic molecules induce apoptosis or necrosis in the target cells, resulting in cell lysis and the release of intracellular enzymes such as lactate dehydrogenase (LDH), which can be measured as a biomarker of cytotoxic activity. Furthermore, the lactase dehydrogenase (LDH) assay (LDH cytotoxicity assay kit, Promega) was conducted using the culture medium to assess cellular damage.

### Statistical analyses

Calculation formulas for determining the glycoforms of CD24 and calculating their contributions as following:

(-Sia CHO-WT): the background of control;

(control) - (CHO-WT): Non-Sia N-, Non-Sia O-, Sia N- and Sia O-;

(-Sia control) - (-Sia CHO-WT): Non-Sia N- and Non-Sia O-;

(nbGFP-PNGF) - (CHO-WT): Non-Sia O- and Sia O-;

(control) - (CHO-WT) - (-Sia control) + (-Sia CHO-WT): Sia N- and Sia O-.

So (-Sia control) - (-Sia nbGFP-PNGF): Non-Sia N-;

(-Sia nbGFP-PNGF) - (-Sia CHO-WT): Non-Sia O-;

(control) - (-Sia control) - (nbGFP-PNGF) + (-Sia nano): Sia N-;

(nbGFP-PNGF) - (CHO-WT) - (-Sia nbGFP-PNGF) + (-Sia CHO-WT): Sia O-.

Among these formulas, (-Sia CHO-WT), (-Sia control) and (-Sia nbGFP-PNGF) are the average flow cytometry fluorescence intensities in Fig. 1I. (CHO-WT), (control), (nbGFP-PNGF) are the average flow cytometry fluorescence intensities in Fig. 1j.

In all cases, the data were presented as means plus standard errors of the mean (SEM). An analysis of the statistical data was performed using the two-tailed student’s test to compare two groups of data. There is a statistical significance for data analysis that has been clarified by *p < 0.05, **p < 0.01 and ***p < 0.001.

GraphPad Prism (v.9) were applied to plot. FlowJo (v.10.8.1) was used to analysis Flow cytometry data. Adobe Illustrator 2020 was applied to compile Figures.

## Supplementary information

Supplementary Materials

Authorship+form

WB raw data

proofig raw data

gating strategies for the flow cytometry data

## Data Availability

All the data during the current study are available within the paper and its Supplementary information, or from the corresponding author upon reasonable request.
