# Supplementary material for: N-deglycosylation targeting chimera (DGlyTAC): a strategy for immune checkpoint proteins inactivation by specifically removing N-glycan
Source: Signal Transduct Target Ther. 2025 Apr 28;10:139. doi: 10.1038/s41392-025-02219-6 (PMC12034804; doi:10.1038/s41392-025-02219-6)
Supplement: Supplementary file 2 — Authorship+form [file 41392_2025_2219_MOESM2_ESM.pdf]

SPRINGER NATURE

Change of authorship request form - Journals (pre-acceptance)

Important information. Please read.

- This form should be used by authors to request any change in authorship (adding/deleting authors) including changes in corresponding authors. This form should not be used for name changes. Please fully complete all sections. Use black ink and block capitals and provide each author's full name with the given name first followed by the family name.

- By signing this declaration, all authors guarantee that the order of the authors are in accordance with their scientific contribution, if applicable as different conventions apply per discipline, and that only authors have been added who made a meaningful contribution to the work.

- Please note, in author collaborations where there is formal agreement for representing the collaboration, it is sufficient for the representative or legal guarantor (usually the corresponding author) to complete and sign the Authorship Change Form on behalf of all authors, **next to the added/removed author(s)**. **(Complete Section 3, followed by Section 6.)**
  In author collaborations where there is no formal agreement for representing the collaboration and **there are more than 10 authors**, one may sign for all, provided the signer appends correspondence that attests that each of the authors have agreed to the change **and the added/removed authors sign the form**. **(Complete Section 3, followed by Section 6.)**

- Please note, we cannot investigate or mediate any authorship disputes. If you are unable to obtain agreement from all authors (including those who you wish to be removed) you must refer the matter to your institution(s) for investigation. Please inform us if you need to do this.

- If you are not able to return a fully completed form within **30 days** of the date that it was sent to the author requesting the change, we may have to withdraw your manuscript. We cannot publish manuscripts where authorship has not been agreed by all authors (including those who have been removed).

- Incomplete forms will be rejected.

- Please return/upload this form, fully completed, to the Journals Editorial Office. The Journal and/or Publisher will consider the information you have provided to decide whether to approve the proposed change in authorship. We may decide to contact your institution for more information or undertake a further investigation, if appropriate, before making a final decision.

SPRINGER NATURE Springer Nature is one of the world's leading global research, educational and professional publishers, created in May 2015 through the combination of Nature Publishing Group, Palgrave Macmillan, Macmillan Education and Springer Science+Business Media.

SPRINGER NATURE

Change of authorship request form - Journals (pre-acceptance)

Section 1: Please provide the current title of manuscript

Manuscript ID no.: SIGTRANS-14748R1

Title: N-deglycosylation targeting chimera (DGlyTAC): a strategy for immune checkpoint proteins inactivation by specifically removing N-glycan

Section 2: Please provide the previous authorship, in the order shown on the manuscript before the changes were introduced. Please indicate the corresponding author by adding (CA) behind the name.

	First name(s)	Family name	ORCID or SCOPUS id, if available
1st author	Li	Li	
2nd author	Jiajia	Wu	
3rd author	Qi	Wu	
4th author	Yaxu	Li	
5th author	Yanrong	Yang	
6th author	Zezhi	Shan	
7th author	Zening	Zheng	
8th author	Xin	Ge	
9th author	Liang	Lin	
10th author	Ping	Wang	

Please use an additional sheet if there are more than 10 authors.

SPRINGER NATURE Springer Nature is one of the world's leading global research, educational and professional publishers, created in May 2015 through the combination of Nature Publishing Group, Palgrave Macmillan, Macmillan Education and Springer Science+Business Media.

SPRINGER NATURE

Change of authorship request form - Journals (pre-acceptance)

Section 3: Please provide a justification for change. Please use this section to explain your reasons for changing the authorship of your manuscript, e.g. what necessitated the change in authorship? Please refer to the (journal) policy pages for more information about authorship. Please explain why omitted authors were not originally included and/or why authors were removed on the submitted manuscript.

During the review and revision of the manuscript, we added Weiqian Cao and Wei Zhang as co-authors as they have managed LC-MS/MS for glycopeptide analysis

Section 4: Proposed new authorship. Please provide your new authorship list in the order you would like it to appear on the manuscript. Please indicate the corresponding author by adding (CA) behind the name. If the Corresponding Author has changed, please indicate the reason under section 3.

	First name(s)	Family name (this name will appear in full on the final publication and will be searchable in various abstract and indexing databases)	Affiliated institute	E-mail address
1st author	Li	Li	Shanghai Tenth People's Hospital	vsign317@163.com
2nd author	Jiajia	Wu	Shanghai Institute of Organic Chemistry	wujiajia_mail@163.com
3rd author	Weiqian	Cao	Institutes of Biomedical Sciences, , Fudan University	wqcao@fudan.edu.cn
4th author	Wei	Zhang	Institutes of Biomedical Sciences, , Fudan University	23211510001@m.fudan.edu.cn
5th author	Qi	Wu	Shanghai Tenth People's Hospital	waiwai@whu.edu.cn
6th author	Yaxu	Li	Tongji University	liyaxu318@163.com
7th author	Yanrong	Yang	Tongji University	1911472@tongji.edu.cn
8th author	Zezhi	Shan	Fudan University Shanghai Cancer Center	18111230016@fudan.edu.cn
9th author	Zening	Zheng	Tongji University	mirrondo@tongji.edu.cn
10th author	Xin	Ge	Shanghai Tenth People's Hospital	xin.ge@tongji.edu.cn

Please use an additional sheet if there are more than 10 authors.

SPRINGER NATURE Springer Nature is one of the world's leading global research, educational and professional publishers, created in May 2015 through the combination of Nature Publishing Group, Palgrave Macmillan, Macmillan Education and Springer Science+Business Media.

SPRINGER NATURE

Change of authorship request form - Journals (pre-acceptance)

Section 3: Please provide a justification for change. Please use this section to explain your reasons for changing the authorship of your manuscript, e.g. what necessitated the change in authorship? Please refer to the (journal) policy pages for more information about authorship. Please explain why omitted authors were not originally included and/or why authors were removed on the submitted manuscript.

During the review and revision of the manuscript, we added Weiqian Cao and Wei Zhang as co-authors as they have managed LC-MS/MS for glycopeptide analysis

Section 4: Proposed new authorship. Please provide your new authorship list in the order you would like it to appear on the manuscript. Please indicate the corresponding author by adding (CA) behind the name. If the Corresponding Author has changed, please indicate the reason under section 3.

	First name(s)	Family name (this name will appear in full on the final publication and will be searchable in various abstract and indexing databases)	Affiliated institute	E-mail address
11th author	Liang	Lin (CA)	Shanghai Institute of Organic Chemistry	lianglin@sioc.ac.cn
12th author	Ping	Wang (CA)	Shanghai Institute of Organic Chemistry	wangp@tongji.edu.cn
13th author				
14th author				
15th author				
16th author				
17th author				
18th author				
19th author				
20th author				

Please use an additional sheet if there are more than 10 authors.

SPRINGER NATURE Springer Nature is one of the world's leading global research, educational and professional publishers, created in May 2015 through the combination of Nature Publishing Group, Palgrave Macmillan, Macmillan Education and Springer Science+Business Media.

SPRINGER NATURE

Change of authorship request form - Journals (pre-acceptance)

Section 5: Author contribution, Acknowledgement and Disclosures. Please use this section to provide a new disclosure statement and, if appropriate, acknowledge any contributors who have been removed as authors and ensure you state what contribution any new authors made (if applicable per the journal or book (series) policy). Please ensure these are updated in your manuscript - after approval of the change(s) - as our production department will not transfer the information in this form to your manuscript.

New acknowledgements:

Funding: This work was supported by grants from the National Key Research and Development Program of China (2022YFC3401500 and 2020YFA0803201 to P.W.), the Major Research Plan of the National Natural Science Foundation of China (Grant No. 92153301 to L.Lin.), the National Natural Science Foundation of China (Grant No. 22177126 to L.Lin.), the National Natural Science Foundation of China (82341028, 31920103007 to P.W.), the Key R&D Projects in Ningxia Hui Autonomous Region (2021BFH03001).
Other: The authors thank Prof. Tingxiu Xiang (The First Afliated Hospital of Chongqing Medical University, China) for generously providing cells, and also thank Ran Xie (The Nanjing University, China) for generously providing plasmids.

New Disclosures (financial and non-financial interests, funding):

The authors declare that they have no competing interests.

New Author Contributions statement (if applicable per the journal policy):

Contributions: P.W., L.Lin and L.L. designed experiments. L.L. as the sole first author of this study performed all the experiments and analyzed the data. The animal studies with the help from J.W., W.C. and W.Z. have managed to LC-MS/MS for glycopeptide analysis, Q.W. and X.D., Y.L. provided method for organizing article images. Y.Y., Z.S. and Z.Z. gave some help to L.L. for use several instruments. L.L. and P.W. wrote the manuscript. All authors have read and approved the article.

State 'Not applicable' if there are no new authors.

SPRINGER NATURE

Change of authorship request form - Journals (pre-acceptance)

Section 6: Declaration of agreement. *All* authors, unchanged, new and removed *must* sign this declaration.
(NB: Please print the form, (docu)-sign and return/upload a scanned copy. Please note that signatures that have been inserted as an image file are acceptable as long as it is handwritten. Typed names in the signature box are unacceptable.) *** Please delete as appropriate. Delete all of the bold if you were on the original authorship list and are remaining as an author.**

	First name	Family name		Signature	Date
1st author	Li	Li	I agree to the proposed new authorship shown in section 4 **/and the addition/removal*of my name to the authorship list** /and the proposed change in corresponding author	Li Li	2025.3.26
2nd author	Jiajia	Wu	I agree to the proposed new authorship shown in section 4 **/and the addition/removal*of my name to the authorship list** /and the proposed change in corresponding author	Jiajia Wu	2025.3.26
3rd author	Weiqian	Cao	I agree to the proposed new authorship shown in section 4 **/and the addition/removal*of my name to the authorship list** /and the proposed change in corresponding author	Weiqian Cao	2025.3.26
4th authors	Wei<br>Liang	Zhang	I agree to the proposed new authorship shown in section 4 **/and the addition/removal*of my name to the authorship list** /and the proposed change in corresponding author<br>Lin (CA)	Wei Zhang	2025.3.26
5th author	Ping<br>Qi	Wu	I agree to the proposed new authorship shown in section 4 **/and the addition/removal*of my name to the authorship list** /and the proposed change in corresponding author	Qi Wu	2025.3.26
6th author	Yaxu	Li	I agree to the proposed new authorship shown in section 4 **/and the addition/removal*of my name to the authorship list** /and the proposed change in corresponding author	Yaxu Li	2025.3.26
7th author	Yanrong	Yang	I agree to the proposed new authorship shown in section 4 **/and the addition/removal*of my name to the authorship list** /and the proposed change in corresponding author	Yanrong Yang	2025.3.26

SPRINGER NATURE Springer Nature is one of the world's leading global research, educational and professional publishers, created in May 2015 through the combination of Nature Publishing Group, Palgrave Macmillan, Macmillan Education and Springer Science+Business Media.

SPRINGER NATURE

Change of authorship request form - Journals (pre-acceptance)

	First name	Family name		Signature	Date
8th author	Zezhi	Shan	I agree to the proposed new authorship shown in section 4 /**and the addition/removal*of my name to the authorship list** /and the proposed change in corresponding author	Zezhi Shan	2025.3.26
9th author	Zening	Zheng	I agree to the proposed new authorship shown in section 4 /**and the addition/removal*of my name to the authorship list** /and the proposed change in corresponding author	Zening Zheng	2025.3.26
10th author	Xin	Ge	I agree to the proposed new authorship shown in section 4 /**and the addition/removal*of my name to the authorship list** /and the proposed change in corresponding author	Xin Ge	2025.3.26

Please use an additional sheet if there are more than 10 authors.

In case of author collaborations with formal agreement:

	Name of consortium/consortia	First name	Family name		Signature	Date
Representative/legal guarantor				I agree to the proposed new authorship shown in section 4 /**and the addition/removal*of my name to the authorship list** /and the proposed change in corresponding author		

Both added/removed authors should complete the information in the first table under Section 6.

SPRINGER NATURE Springer Nature is one of the world's leading global research, educational and professional publishers, created in May 2015 through the combination of Nature Publishing Group, Palgrave Macmillan, Macmillan Education and Springer Science+Business Media.

	First name	Family name		Signature	Date
11th author	Liang	Lin	I agree to the proposed new authorship shown in section 4 /**and the addition/removal*of my name to the authorship list** /and the proposed change in corresponding author	Liang lin	20253.26
12th author	Ping	Wang	I agree to the proposed new authorship shown in section 4 /**and the addition/removal*of my name to the authorship list** /and the proposed change in corresponding author	Ping Wang	20253.26

Please use an additional sheet if there are more than 10 authors.

In case of author collaborations with formal agreement:

	Name of consortium/consortia	First name	Family name		Signature	Date
Representative/legal guarantor				I agree to the proposed new authorship shown in section 4 /**and the addition/removal*of my name to the authorship list** /and the proposed change in corresponding author		

Both added/removed authors should complete the information in the first table under Section 6.

---- End of form ----

SPRINGER NATURE Springer Nature is one of the world's leading global research, educational and professional publishers, created in May 2015 through the combination of Nature Publishing Group, Palgrave Macmillan, Macmillan Education and Springer Science+Business Media.
