# Supplementary material for: N-deglycosylation targeting chimera (DGlyTAC): a strategy for immune checkpoint proteins inactivation by specifically removing N-glycan
Source: Signal Transduct Target Ther. 2025 Apr 28;10:139. doi: 10.1038/s41392-025-02219-6 (PMC12034804; doi:10.1038/s41392-025-02219-6)
Supplement: Supplementary file 4 — proofig raw data [file 41392_2025_2219_MOESM4_ESM.pptx]

## Slide 1
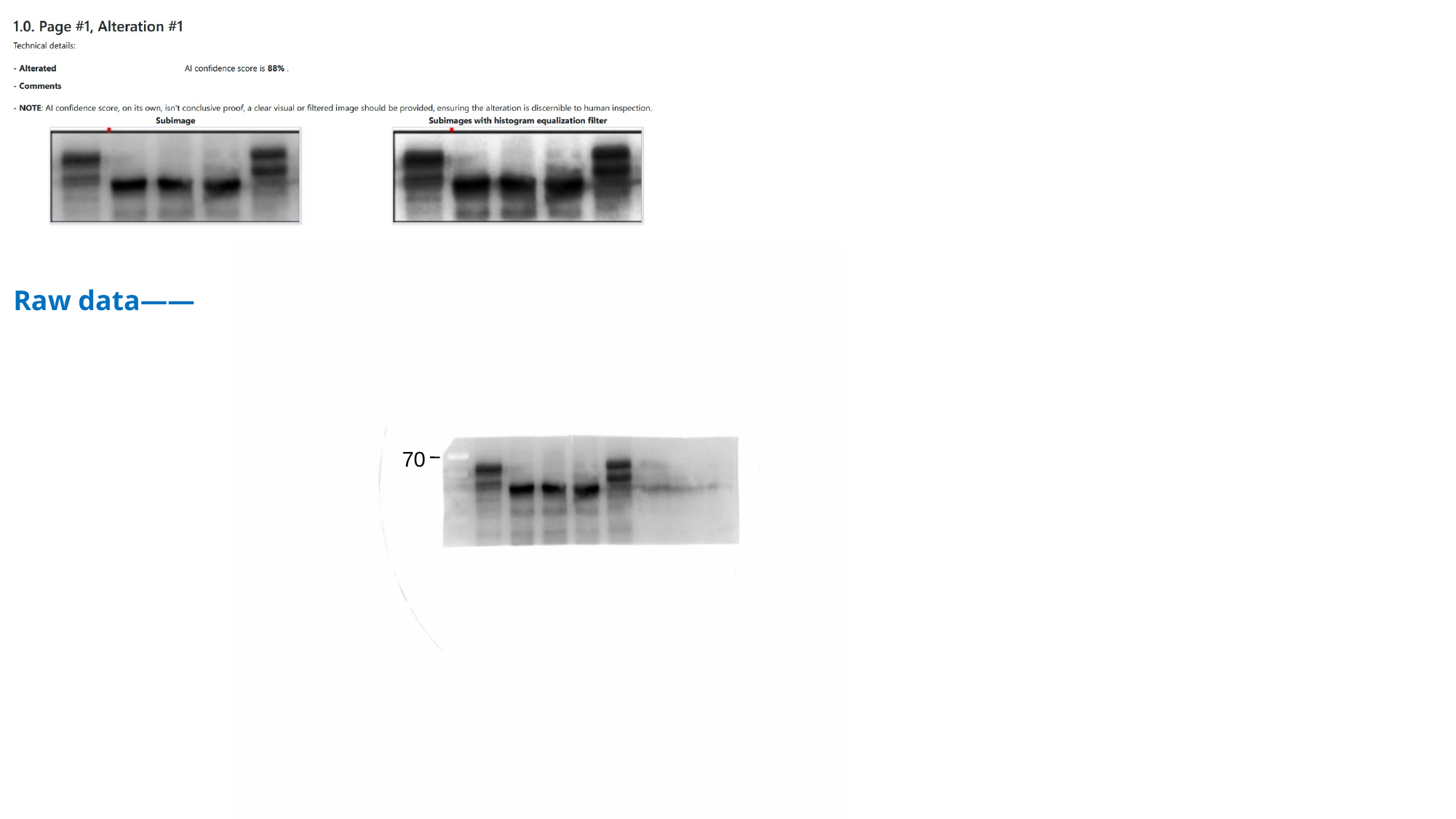

70
Raw data——

## Slide 2
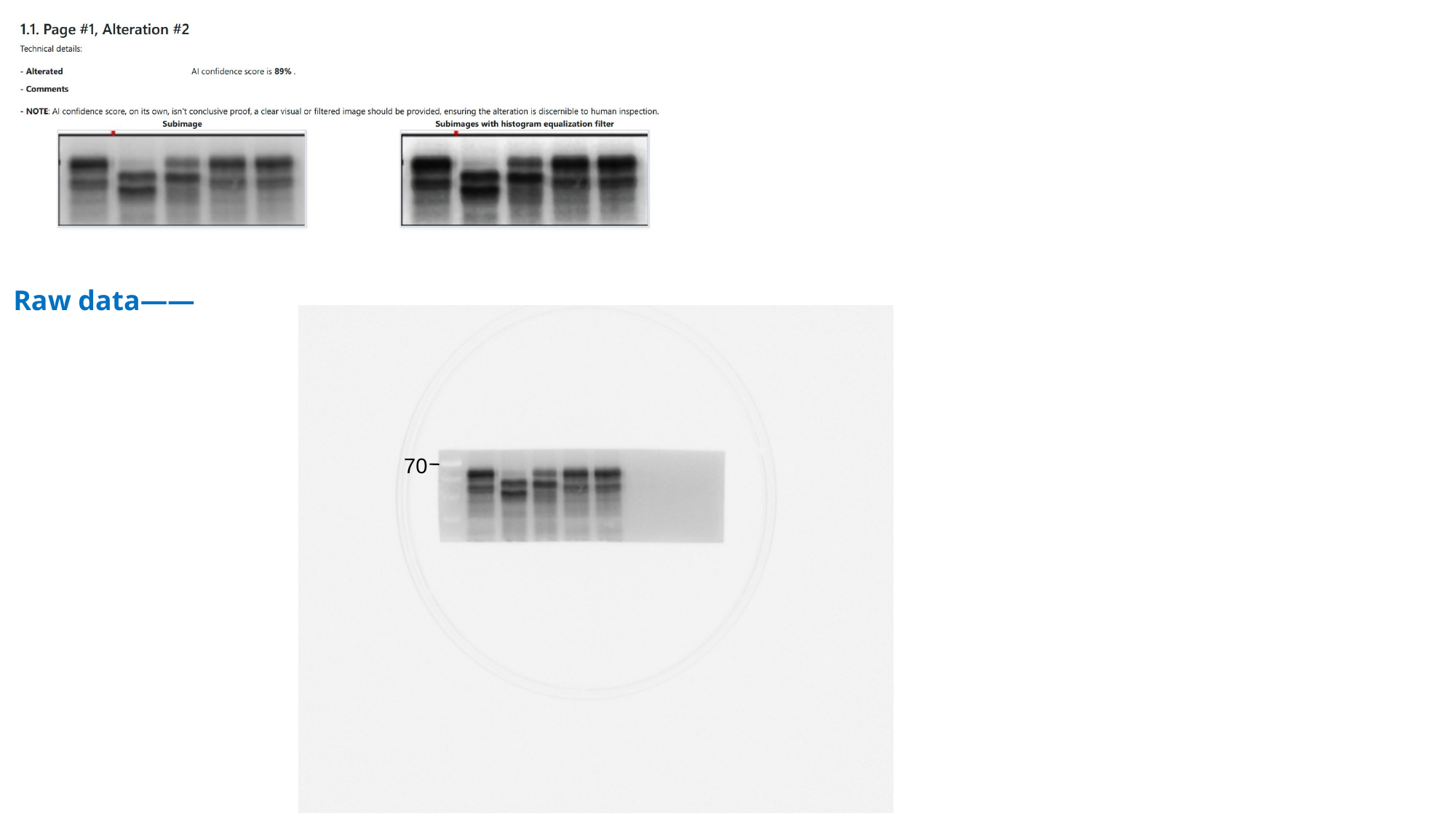

Raw data——
70

## Slide 3
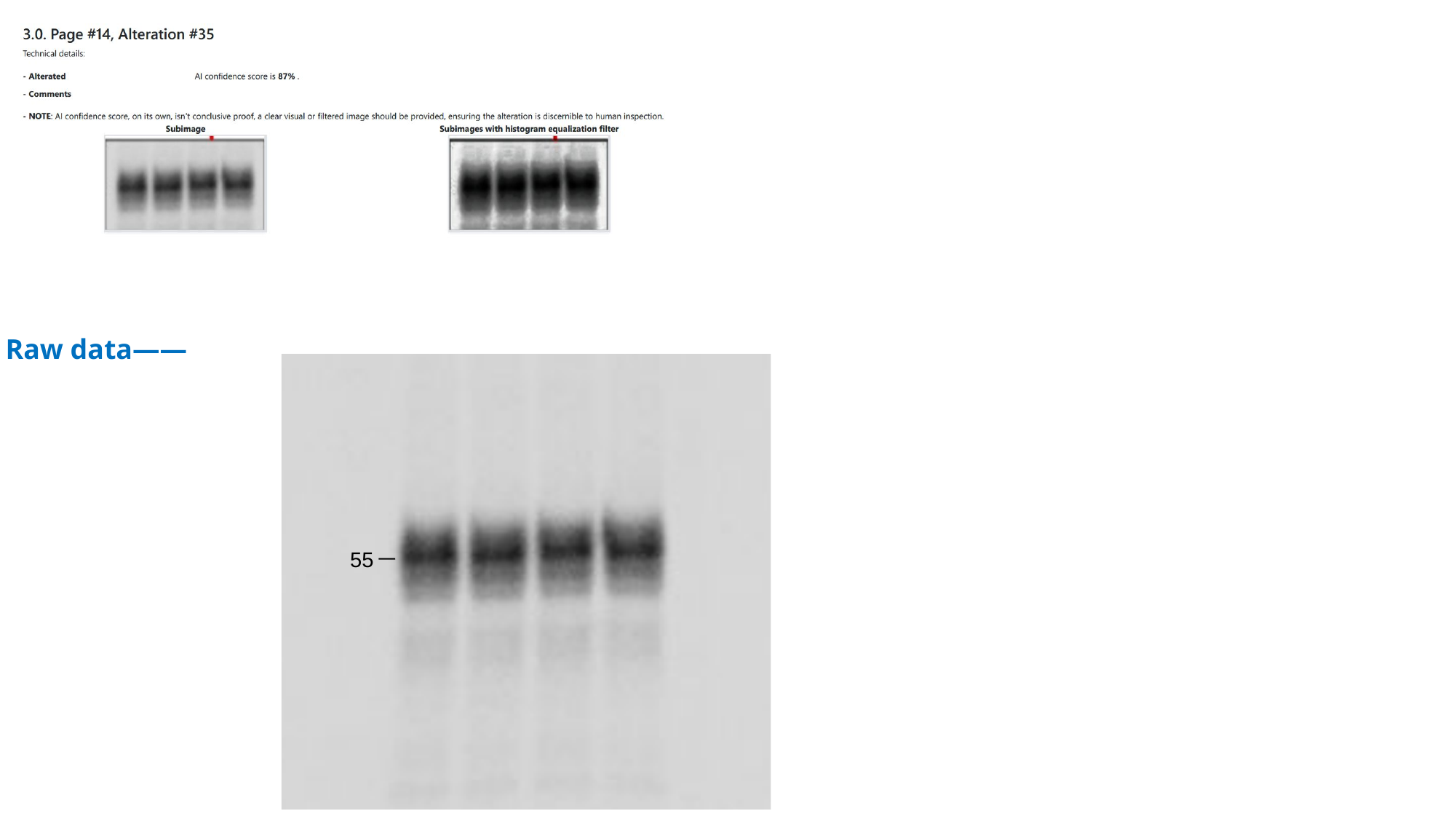

Raw data——
55

## Slide 4
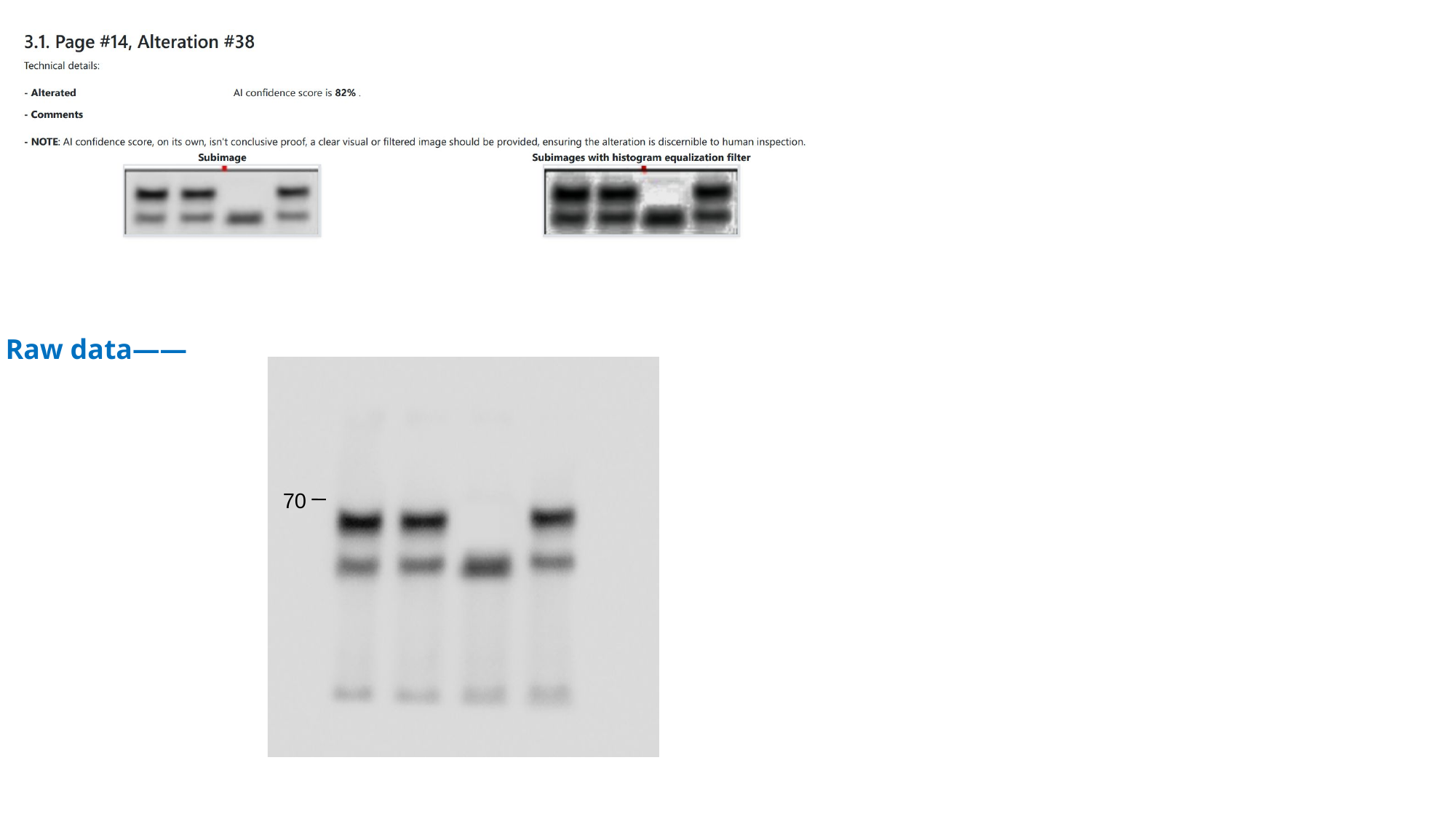

Raw data——
70

## Slide 5
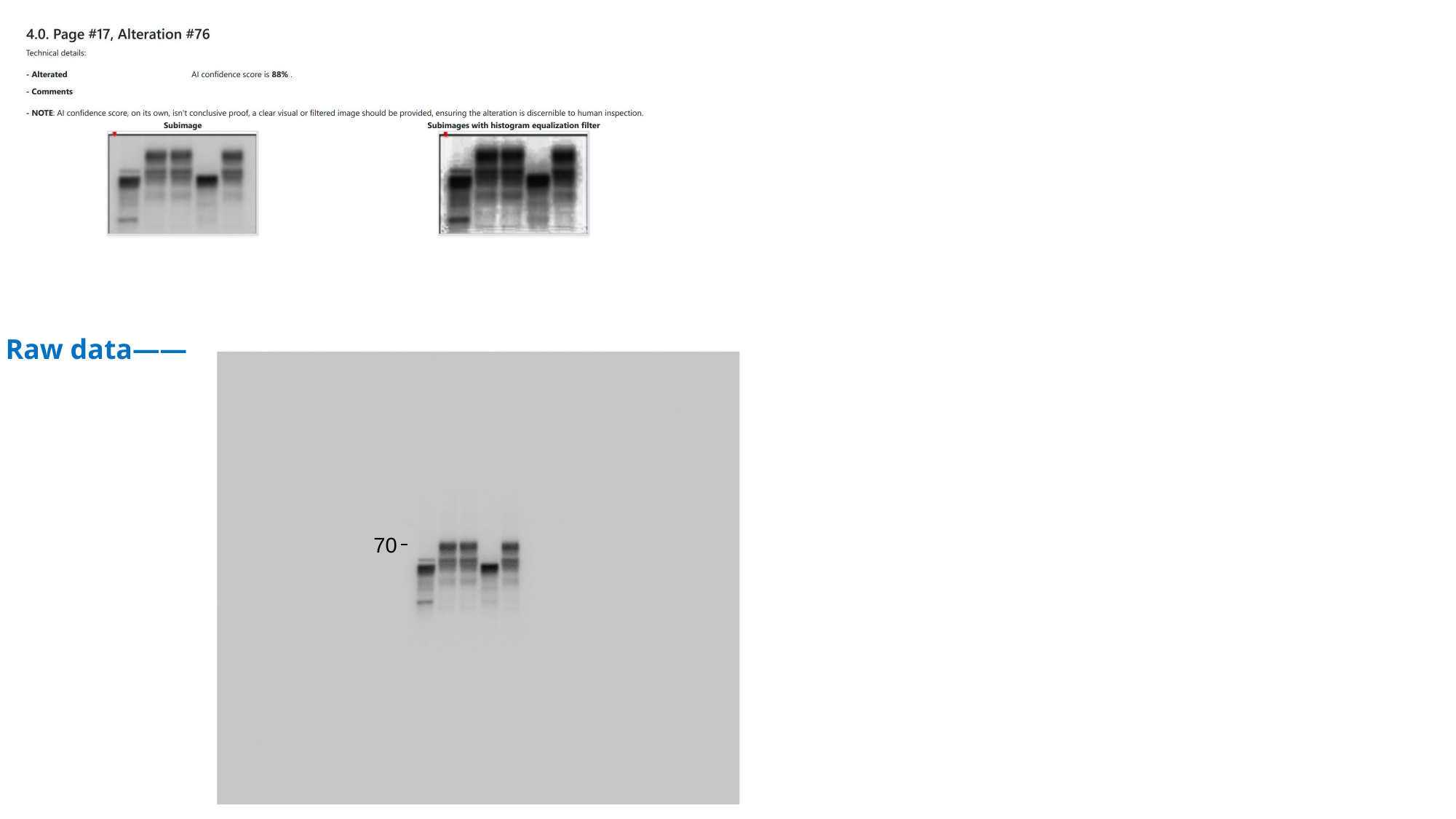

Raw data——
70

## Slide 6
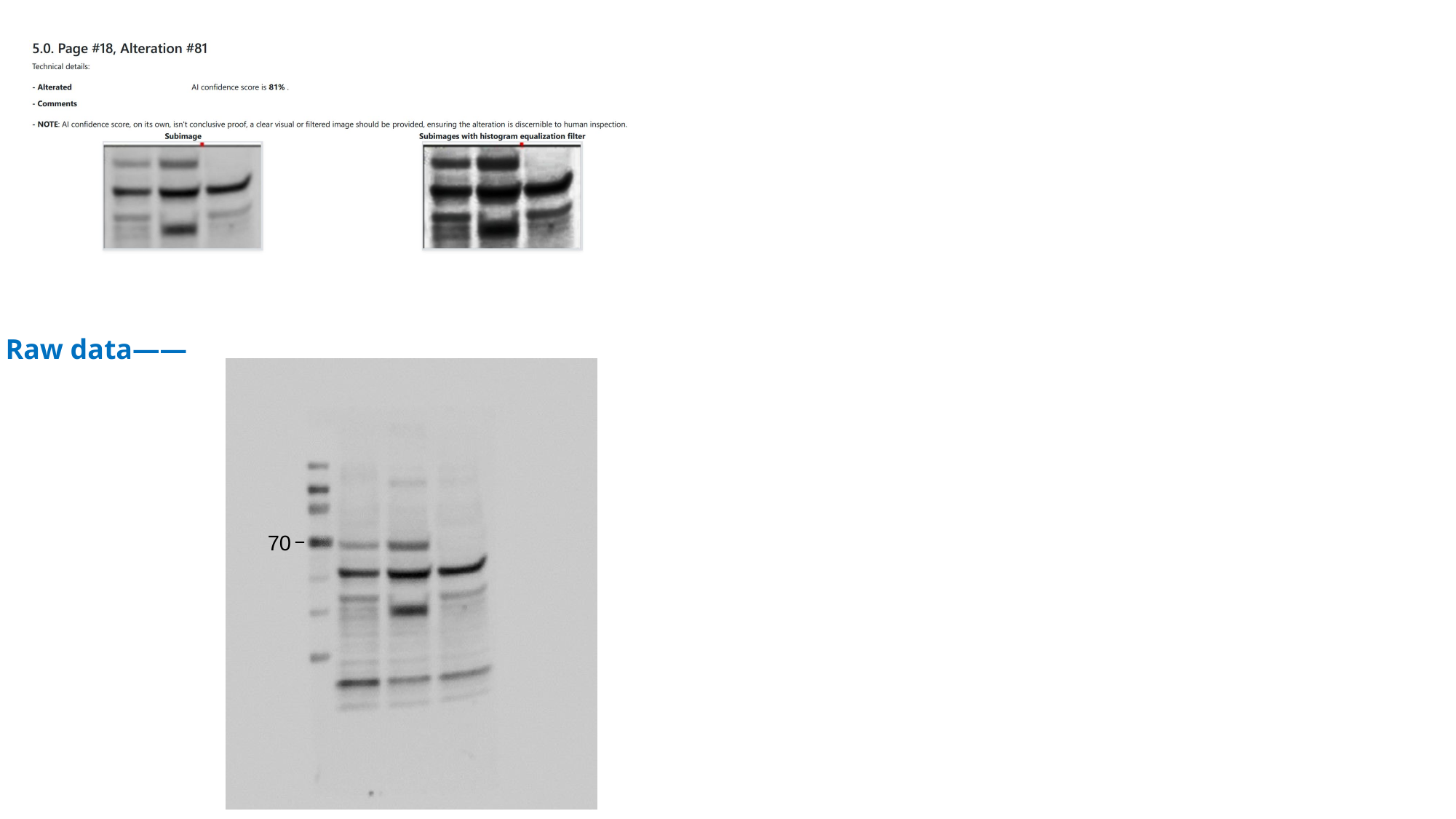

Raw data——
70

## Slide 7
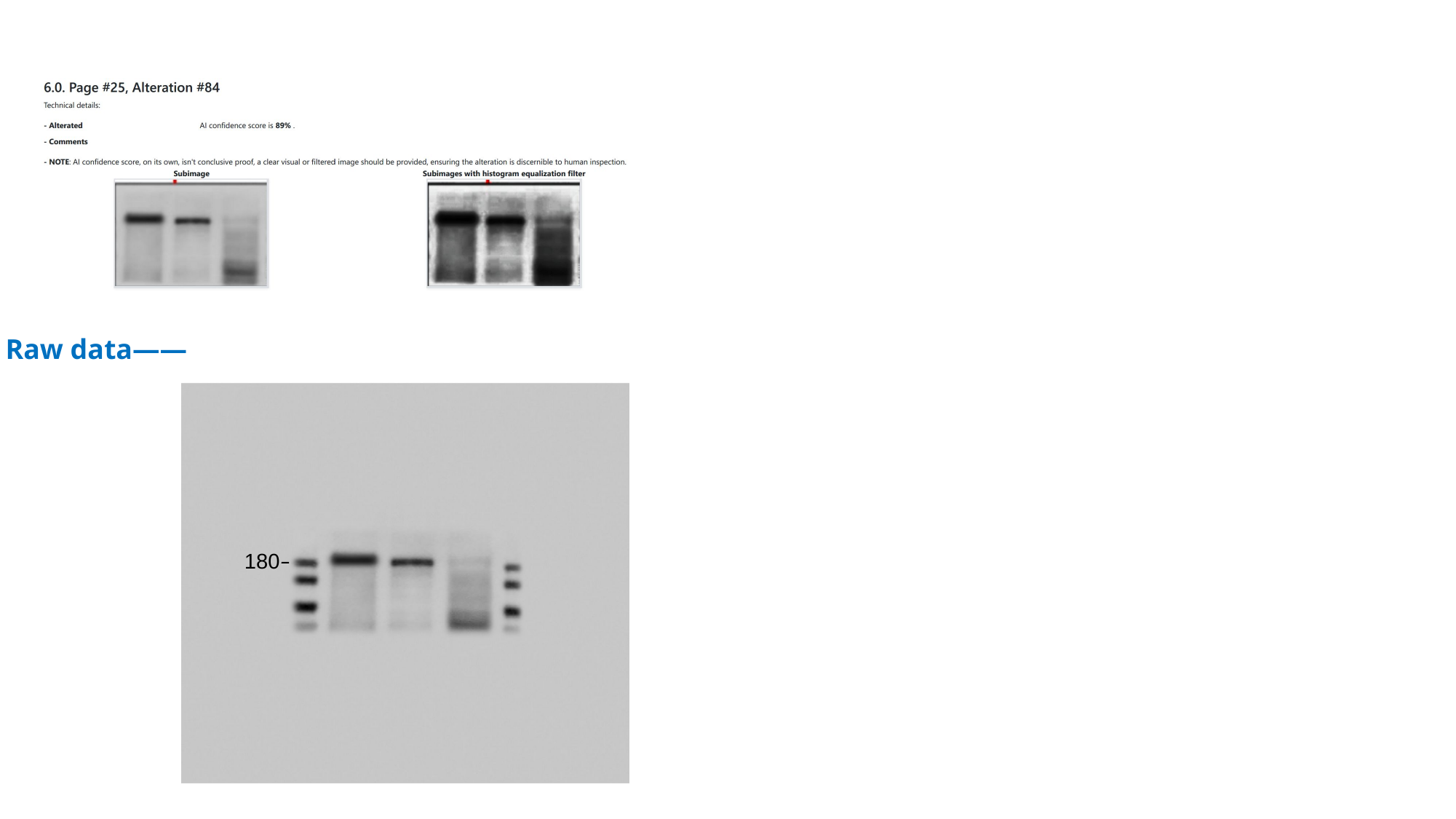

Raw data——
180

## Slide 8
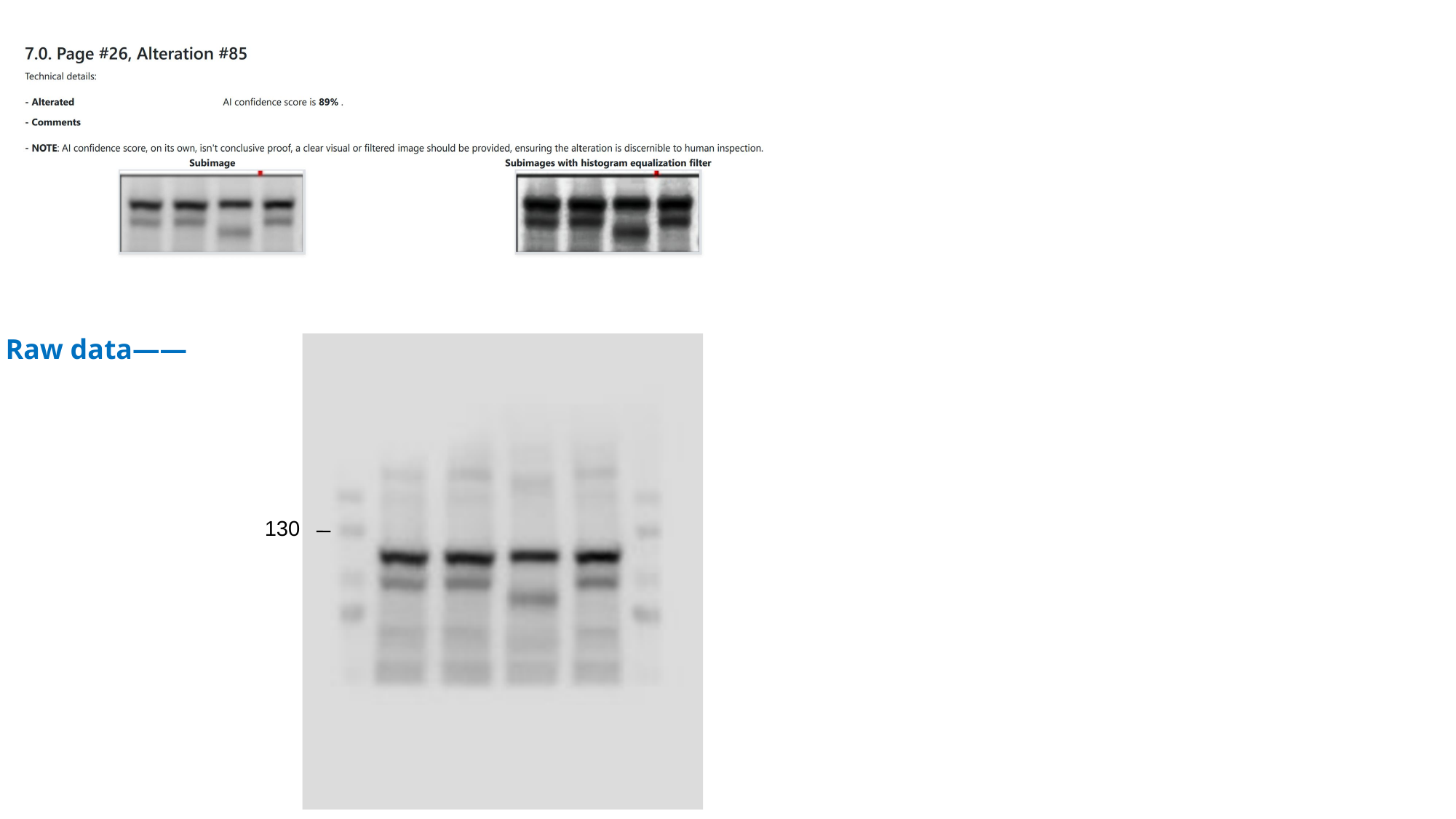

Raw data——
130

## Slide 9
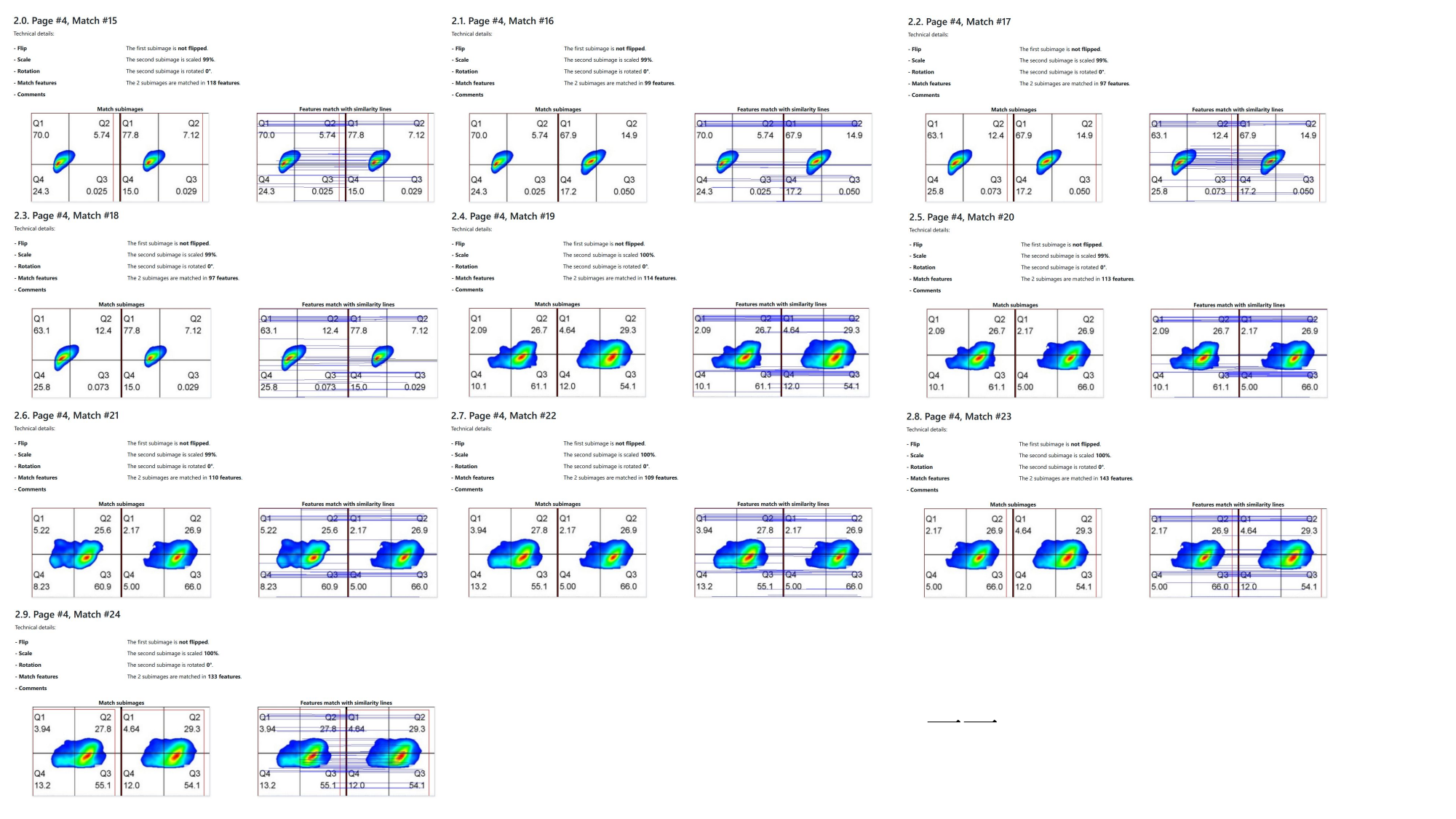

对指出的所有流式图一一进行了核实，没有图像是重复使用的
