# Supplementary material for: N-deglycosylation targeting chimera (DGlyTAC): a strategy for immune checkpoint proteins inactivation by specifically removing N-glycan
Source: Signal Transduct Target Ther. 2025 Apr 28;10:139. doi: 10.1038/s41392-025-02219-6 (PMC12034804; doi:10.1038/s41392-025-02219-6)
Supplement: Supplementary file 5 — gating strategies for the flow cytometry data [file 41392_2025_2219_MOESM5_ESM.pptx]

## Slide 1
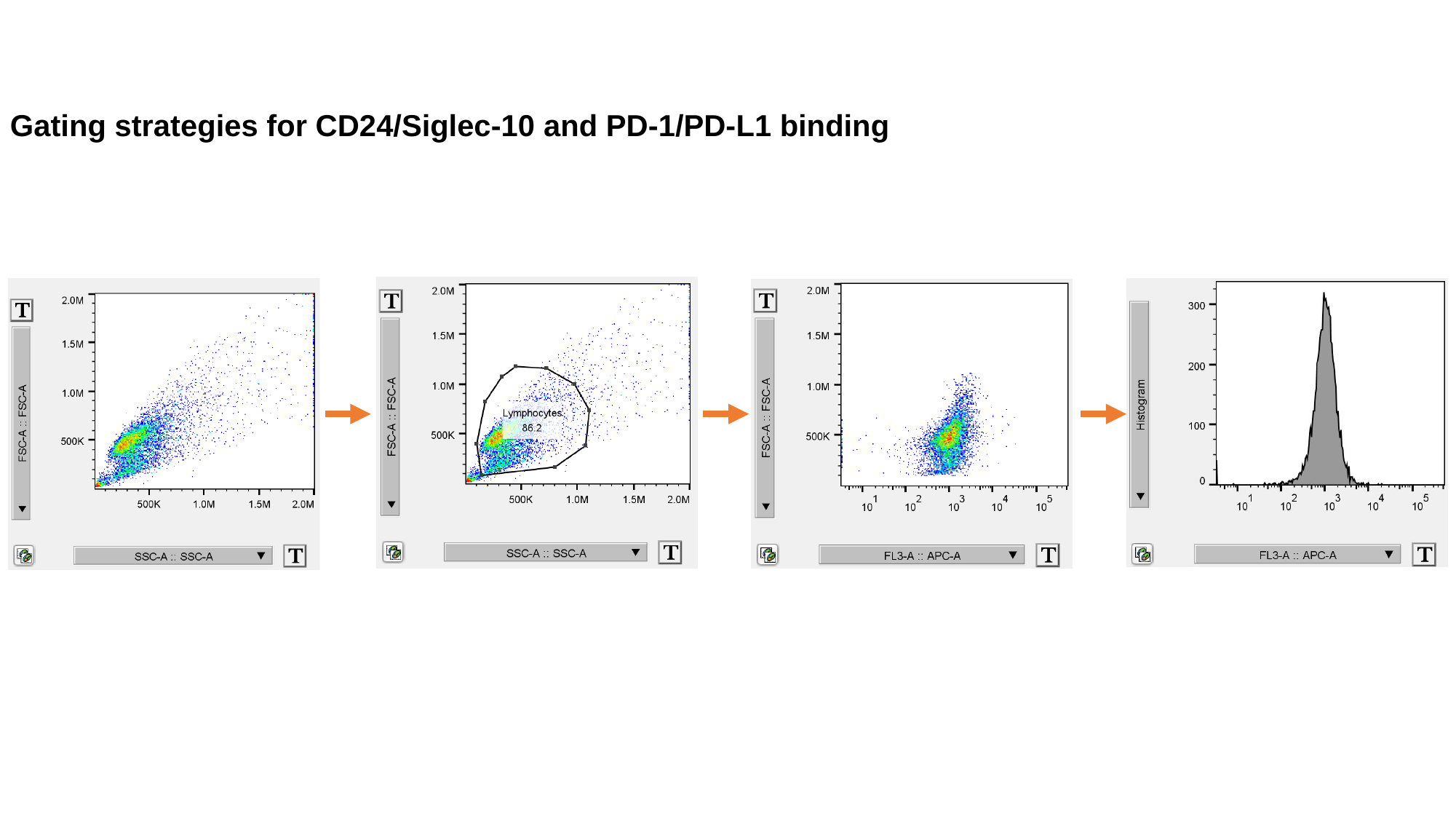

Gating strategies for CD24/Siglec-10 and PD-1/PD-L1 binding

## Slide 2
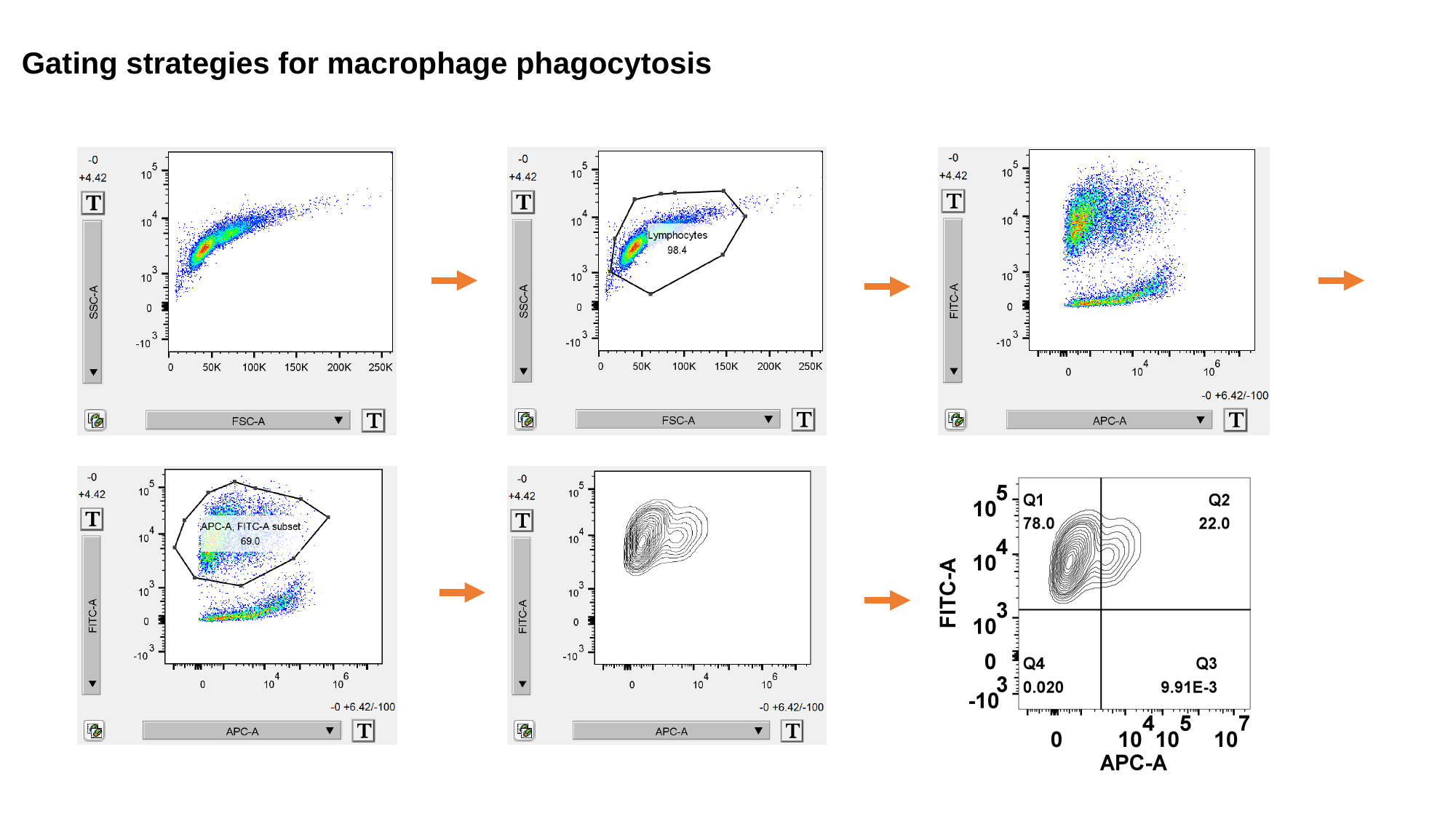

Gating strategies for macrophage phagocytosis

## Slide 3
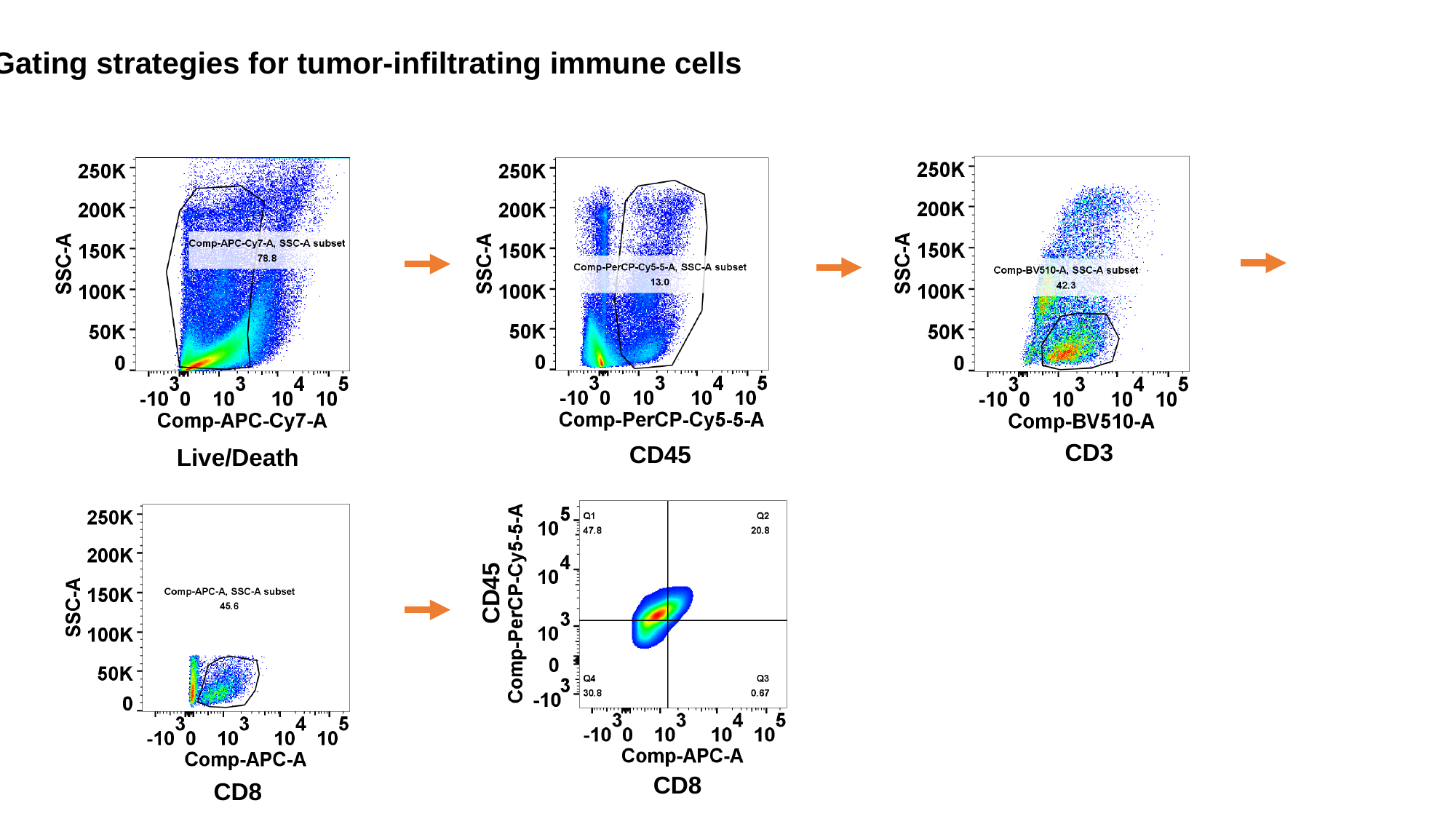

Gating strategies for tumor-infiltrating immune cells
CD3
CD45
Live/Death
CD45
CD8
CD8
